# *cpm*: A python library for theory-driven modelling in computational psychiatry

**DOI:** 10.1371/journal.pcbi.1014481

**Published:** 2026-07-13

**Authors:** Lenard Dome, Frank H. Hezemans, Kenza Kadri, Ben J. Wagner, Andrew Webb, Tobias U. Hauser

**Affiliations:** 1 Department of Psychiatry and Psychotherapy, Faculty of Medicine, University Tübingen, Tübingen, Germany; 2 German Center for Mental Health (DZPG), Tübingen, Germany; 3 Max Planck Institute for Biological Cybernetics, Tübingen, Germany; 4 Institute of Cognitive Science, Osnabrück University, Osnabrück, Germany; 5 Max Planck UCL Centre for Computational Psychiatry and Ageing Research, University College London, London, United Kingdom; Brown University, UNITED STATES OF AMERICA

## Abstract

The Computational Psychiatry Modelling (*cpm*) toolbox is a Python library for theory-driven modelling in computational psychiatry and cognitive (neuro-)science. *cpm* integrates a wide range of established approaches into a single framework. It is designed to be accessible to both expert and non-expert modellers in order to conduct cutting-edge computational modelling, while adhering to best practices. The toolbox provides a flexible, modular architecture that adjusts to different needs. It covers a wide range of problems (such as risky decision-making, reward/punishment learning, perceptual metacognition), models (including those based on associative, reinforcement learning, and signal detection theories), and methods (such as hierarchical parameter estimation using empirical and variational Bayesian techniques). Such a customisable toolbox aims to lower the barrier for beginners and to facilitate access to advanced modelling approaches in psychiatry and beyond.

## Author summary

Computational psychiatry is a field that makes extensive use of computational methods to understand mental health. In this field and related disciplines, there is a strong need to design, implement, and apply mathematical models of behaviour and cognition (such as reinforcement learning or Bayesian models). However, coding these models from scratch poses a significant challenge for researchers without extensive computational training, including clinicians and other stakeholders who are interested in understanding the underlying principles of mental disorders. Here, we describe the *cpm* toolbox, providing a step-by-step walkthrough of the current design, alongside links to related resources. We believe that building such an overarching, customisable, and user-friendly toolbox can facilitate access to computational modelling, and thus jump-start computational approaches in the field and beyond.

## Introduction

Computational approaches now play a central role in cognitive neuroscience and psychiatry research, advancing our understanding of cognition and its disruption in psychiatric disorders [1–5]. Computational models are compelling as they are not just statistical summaries, but formulate a generative process [6,7]. They embed hypotheses about how brain processes operate, how mechanisms of cognition take shape, and how interactions between disparate processes produce behaviour. In essence, they are theories of cognition, brain, and behaviour. This formalism enables a deep and mechanistic understanding of how psychological processes operate differently in various mental illnesses.

One key advantage of using these computational models is that they require the researcher to unambiguously express their theories in terms of mathematical formalisms, but in return facilitate testing those theories against empirical observations. This approach opens up a fruitful path forward in understanding intricate cognitive abilities in a broad sense and psychopathology in particular [1,5,6,8–10]. They specify the generative mechanics that link psychological processes to observable outcomes (e.g., behaviour) [6,11]. Theory-driven computational psychiatry is the embodiment of these ideas, and because of this, the field puts computational models front and centre, thus creating a complex, rapidly advancing space in an attempt to understand psychiatric conditions.

Despite the rapid growth and success [12–15] of computational psychiatry in the last decade, the field still faces several limitations. First, modelling practices remain fragmented and lack standardisation: Similar models tend to be implemented differently across laboratories, often with unstated theoretical assumptions and limited shared code and documentation. Such heterogeneity can lead to inconsistent results, in some cases driven by undetected bugs in code [16] which may propagate as new generations of researchers build on existing implementations. Although the importance of standardisation is increasingly emphasised [17,18], consistent adoption remains limited. Second, as an inherently interdisciplinary field, computational psychiatry presents practical challenges for researchers with limited computational expertise [19], including clinicians and experimental researchers. Implementing and validating computational models requires substantial technical knowledge and skills, and gaps in this expertise can lead to erroneous or suboptimal analysis of valuable clinical and behavioural data.

We propose that providing a general-purpose framework can address several of these challenges and facilitate robust and accessible computational modelling. More broadly, the creation of standardised workflows and readily available resources has been crucial to the advancement of numerous disciplines, often acting as a catalyst propelling an entire field forward. For example, the widespread adoption of neuroimaging methods (such as functional MRI) was closely linked to the development of toolboxes such as SPM [20,21] and FSL [22]. Similarly, progress in machine learning was critically linked to open access libraries, such as scikit-learn [23], Tensorflow [24], and PyTorch [25]. In computational psychiatry, this level of standardisation is already evident for many data-driven approaches [26,27], which are supported by mature software ecosystems (e.g., scikit-learn [23] for predictive modelling and classification; PCNToolkit [28] for normative modelling of neuroimaging data). In contrast, for theory-driven, mechanistic modelling approaches [29], implementations remain more heterogeneous, with fewer general-purpose tools supporting end-to-end analysis workflows.

We argue that the time has come for a unified modelling methodology in theory-driven computational psychiatry, because the best practices are now relatively clearly formulated [18] and the field is beginning to converge to a limited number of frequently used computational models for canonical tasks (such as the “two-step” task [30], “affective go/no-go” task [31], and perceptual metacognition paradigms [32]). This convergence has been facilitated by the availability of openly shared task implementations, enabling large-scale data collection across populations and laboratories [33].

Here, we present the Computational Psychiatry modelling (*cpm*) toolbox, an easy-to-use Python library that allows researchers to develop and apply computational models and to leverage a wide variety of well-established methods, without the need for in-depth technical expertise. We believe that such an open-source, open-access toolbox will help facilitate and harmonise computational modelling approaches in computational psychiatry and its adjacent fields. Although *cpm* is framed around computational psychiatry, its methods and models are broadly applicable across psychology and cognitive neuroscience more generally. This reflects the fact that computational psychiatry relies on established models and inference techniques originally developed in these fields, which are then applied to formalise latent mechanisms that can be mapped to psychiatric symptoms, groups, and clinical outcomes.

### Scope

*cpm* is a modular toolbox designed to cover a wide variety of tasks and models often used in computational psychiatry (and its non-clinical sibling, cognitive neuroscience); with its scope intentionally bounded around theory-driven cognitive modelling.

The toolbox can accommodate many different models due to its customisable modelling interface, but is designed to focus on theory-driven cognitive models that specify the mechanistic generative process linking trial-level inputs to trial-level predictions and latent states. In this context, the “model” typically requires some trial-level information, such as what stimulus is presented to the participant, and a process that maps this input to some observable outcomes, such as choices between two alternative stimuli. In the current version, we prioritise model families that are widely used in computational psychiatry and which can be mathematically expressed at the trial level. Currently, *cpm* provides metacognitive sensitivity estimators, reinforcement-learning-style and value-based decision-making models. For example, the toolbox offers multiple models based on Prospect Theory [34]. Future releases will expand the toolbox to include additional model categories, such as models of effort-based decision-making [35].

The models currently included in *cpm* were selected according to four criteria. First, they cover modelling traditions widely used in computational psychiatry. Second, they span more than one theoretical tradition rather than concentrating within a single model family, with current coverage including reinforcement learning and prospect-theoretic models. Third, they target areas not already well served by existing toolboxes; evidence-accumulation models, for instance, are extensively covered elsewhere, and we deliberately do not duplicate this coverage (see Related work for a detailed comparison). Fourth, the included models have established empirical use cases, allowing new users to reproduce familiar analyses as they learn the framework. Model prioritisation is guided by community feedback, ensuring the toolbox remains responsive to user needs. Beyond implementing models, the toolbox is built around a standard interface in which a model takes in trial-level information and outputs some predictive quantity. Constraining the model interface in this way puts emphasis on running model families through the same trial-by-trial workflow, so researchers can reuse the same code across models and make minimal changes to only those parts of the model that are of interest.

### Guide to the paper

In this paper, we address both non-expert and more technically minded researchers, with the goal of supporting researchers from diverse backgrounds in applying computational modelling and contributing to the toolbox. Therefore we have structured the paper such that it can be read from multiple entry-points. Readers primarily interested in using *cpm* can begin with a tutorial walkthrough in the Results section that demonstrates the application of the toolbox for a minimal modelling loop. Technical readers interested in more implementation-level, architectural details, or contributing to the open codebase of the toolbox, can proceed to the Design and implementation and Implementation sections, which outline the architectural decisions and intended extension points.

## Design and implementation

### Design principles

The development of *cpm* is guided by scientific considerations of what makes computational modelling useful and trustworthy in practice. As a result, a modelling toolbox should not merely enable the effective estimation of parameters, but should actively support the complete modelling cycle: specifying assumptions, generating predictions, comparing model output to empirical data, comparing multiple models on data, and evaluating models through various recovery-type procedures.

*cpm* is built to make theoretical assumptions explicit and to enforce standard practices in both model building and parameter estimation. Whenever possible, we focus on design elements that encourage directly mirroring mathematical specifications, enforcing the specification of priors and parameter bounds. This transparency is intended to reduce the risk of results differing due to inconsistent choices in defining parameters, hidden implementation details, and undocumented modelling choices [16,18]. Therefore, the central goal of *cpm*’s framework is to reduce analytical degrees of freedom, which includes inconsistent preprocessing, reporting conventions, and likelihood implementations, while preserving the flexibility to express different theoretical commitments. The toolbox pursues this balance by standardising workflows and building modular interfaces between components, rather than only offering a catalogue of models. This design aims to make results more comparable across studies and groups while keeping the space of possible models open.

Scientific progress can often be driven by fast iterations: implementing several variants within the same model family, simulating qualitative behaviour, testing model parameter and model identifiability, and comparing against alternatives. *cpm* thus attempts to lower the friction of moving between these steps by exposing reusable components for model construction and parameter estimation. It does so via flexible interfaces that contribute to the plug-and-play user experience. This modularity makes exploring model variants accessible and cost-effective while maintaining consistent workflows.

In the remainder of this section, we will discuss the overarching design principles that guide the implementation of *cpm* (modularity, reproducibility, and openness to contribution), and will leave more technical details of the specifics of the design to a later section (Implementation).

#### Modularity.

**Fig 1:**
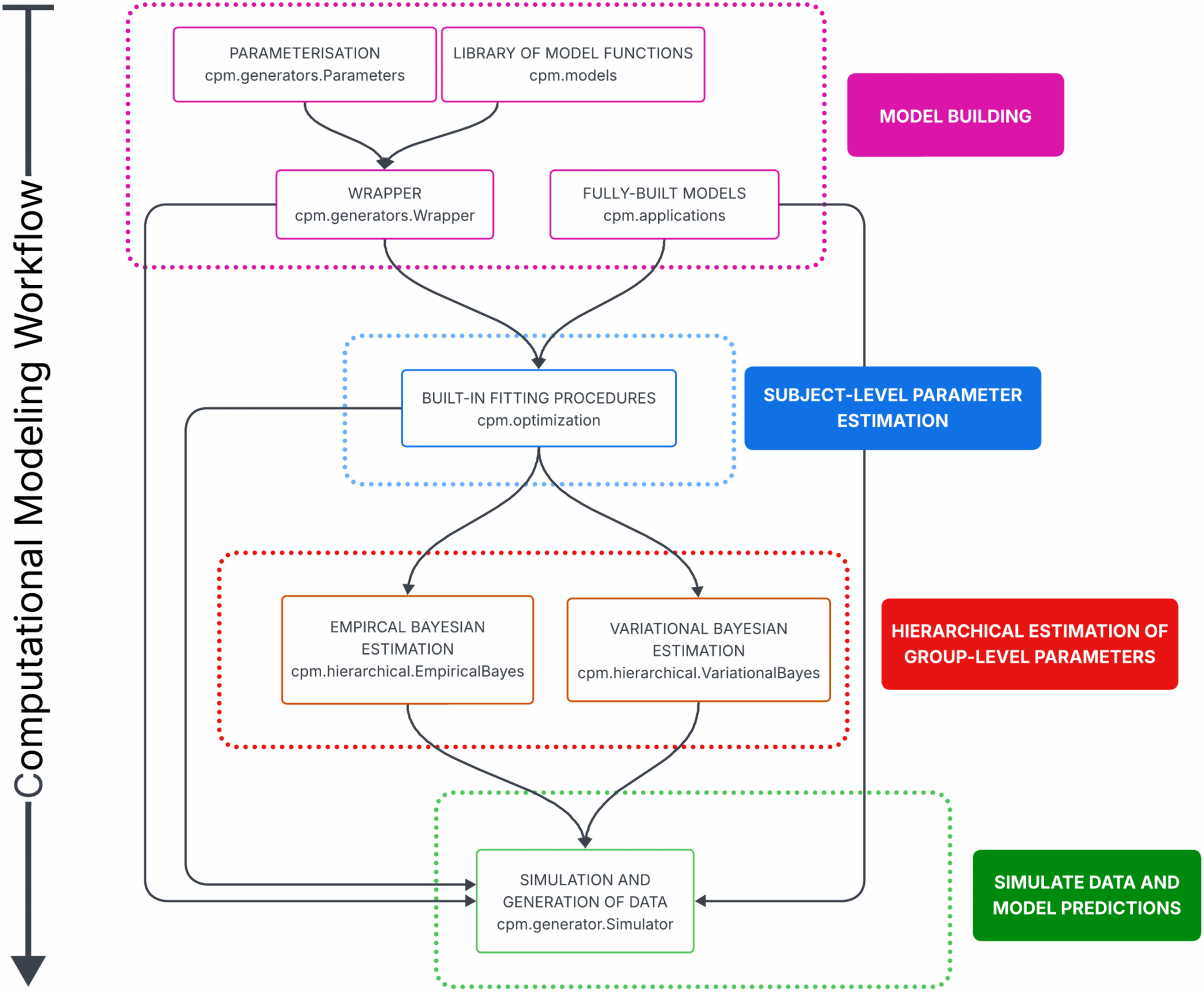
This figure illustrates how various toolbox components (rectangular text boxes) relate to one another. Arrows indicate direction, with an arrow from *WRAPPER* to *BUILT-IN FITTING PROCEDURES* signifying that optimization methods can utilize wrapper objects. Colours differentiate feature groups (dotted enclosures).

Modularity is a design principle in software engineering where a program is split into separate, self-sufficient pieces, called modules, that can be developed and replaced independently. It is a core design principle of *cpm*, ensuring that individual components can be developed, tested, and reused independently, and that researchers can engage with the toolbox at the level that best suits their needs. The toolbox is designed to separate primitive model components, orchestration layers, application-level model presets, parameter-estimation routines, and hierarchical estimation methods. Primitive model components (e.g., learning rules, competitive attentional gating) form the building blocks researchers combine when implementing a model. Orchestration layers (e.g., cpm.generators) handle the mechanics of running a model over trial sequences and managing parameters – the equivalent of writing a simulation loop. Parameter-estimation routines map directly to the model-fitting step, while hierarchical estimation methods extend this to group-level inference. Application-level presets package common model implementations for researchers who want a ready-made starting point rather than building from scratch. This modularity allows users to engage with the toolbox exactly where it best serves their modelling needs. Fig 1 shows one of the simplest feed-forward workflows that is implemented using only pre-defined modules of *cpm*. For the implementation-level organisation of modules, see Implementation section. For an overview of the Python module structure, see Table 1. Although we present a modelling workflow as a fixed feed-forward process for clarity (indicated by the large arrow on the left side), we recognise that modelling is frequently more complex. For instance, workflows that implement model evaluation prior to data collection, such as parameter or model recovery, do not align with the type of workflow illustrated here. However, due to the modular design, *cpm* can accommodate them with ease and without ever leaving the toolbox.

**Table 1:** Overview of CPM toolbox modules.

Module	Purpose	Key Features
cpm.models	Model definitions for learning, decision-making, etc.	Implements core components of computational model components (e.g., Delta Rule, Q-Learning Rule, decision policies, activation functions).
cpm.generators	Simulation and parameter management.	Classes for running models (Wrapper, Simulator), handling parameters, and organizing trial data.
cpm.optimisation	Model fitting and parameter estimation with built-in parallelisation and automated for subject-level estimates.	Loss/objective functions (log-likelihoods, cross-entropy), optimisation algorithms (Bayesian, genetic, gradient-based).
cpm.hierarchical	Hierarchical parameter estimation.	Empirical and variational Bayesian methods for joint estimation of subject-level parameters and their group-level means and variances.
cpm.applications	Ready-to-use implementations of specific models or analysis methods	Pre-built end-to-end pipelines combining models, generators, and wrappers for common problems (e.g., reinforcement learning, signal detection).
cpm.datasets	Example datasets from common canonical experimental tasks.	Built-in datasets to test your models while building them.
cpm.utils	Miscellaneous utilities	Helper functions for data manipulation, metadata, and general-purpose tasks.

This design philosophy extends to macro tasks as well, such as implementing custom models that are not provided pre-built. In the toolbox’s framework, users are only required to specify the computations of their model on a given trial. The toolbox then handles the implementation details required to evaluate the model: iterating over trials, computing log-likelihoods, managing edge cases and organizing outputs. More specifically, this design removes the need for writing nested loops or deciphering cryptic code. Rather, users only need to match the mathematical specification they intend to follow. Therefore, the reduced need to depend on user code for tasks unrelated to the theoretical specification of models allows us to focus on the interfaces between these layers and steps in the workflow. Basic interfaces between these layers allow an efficient interaction between components and the multiple entry-points to the workflow contribute to a plug-and-play user experience without sacrificing customisability. This approach is sometime referred to as Polymorphism in programming.

#### Reproducibility utilities.

Reproducible modelling starts with a clear and machine-readable representation of trial-level information. In combination with the enforcement of transparent modelling practices, such as defining priors, exporting important details about the optimisation routines, and reporting modelling decisions, *cpm* both encourages and contributes to reproducible end-to-end workflows.

Archival utilities are built in to promote reproducible workflows. For example, export functions accompany both parameter management systems and optimisation procedures. They are designed to record additional information about parameters and optimisation routines, and convert this into tabular data format that can be saved as a plain text file. Parameter exports support outputting bounds and prior specifications, while optimisation routines likewise record extensive metadata, including warnings, Hessian matrices, goodness-of-fit metrics, iteration counts, and termination criteria. More generally, *cpm* stores metadata throughout the workflow wherever possible, so analyses can be inspected, shared, and rerun with minimal ambiguity.

### Implementation

The features of the toolbox are organised into a number of sub-modules, each of which has a distinct purpose in the computational modelling workflow. Table 1 presents these sub-modules with brief descriptions. Most workflows can be implemented by different arrangements and reuse of these sub-modules (see Fig 1 for one possible instantiation of a minimal workflow). This organisation into sub-modules directly reflects and supports the modular design of *cpm*. Novel workflows can thus be implemented without needing to rewrite significant portions of the software architecture, and only requires introducing a small number of specific features into the codebase.

The core workflow in *cpm* follows a simple pipeline that maps to the sub-modules: task data are loaded and formatted, a model is specified, the model generates predictions via simulation, an objective function. An objective function is a general term to define any function that the optimisation algorithm tries to minimise or maximise. Log-likelihood functions, which are most often used in computational psychiatry, are a specific type of objective functions. links predictions to observations, and parameters are fit by optimisation. This core workflow can be broken down and supplemented to estimate group-level parameters through hierarchical estimation techniques from cpm.hierarchical; or completely broken down and rearranged to create parameter recovery using nothing but cpm.generators and cpm.optimisation. This modular way of implementing workflows results in some modules becoming core building blocks, while others remain optional extensions. The cpm.generators and cpm.optimisation sub-modules are probably the most often recurring parts of the workflows, whereas functionalities such as cpm.models might be less frequent for some applications. One reason for this is the separation of core model building blocks (reusable components for model building, cpm.models) from ready-to-use applications and wrapper functions for fully-custom code. Experienced users will likely focus on these core modules, while cpm.applications and cpm.datasets act as an on-ramp and reference implementation for common workflows.

#### Core components.

Model construction in *cpm* is organized around a small set of reusable computational “building blocks” that form the foundation for cognitive models and larger workflows. These are pre-implemented, widely-used equations that correspond to computational or mathematical representations of psychological processes, such as error-driven learning [36] or the SoftMax decision rule [37]. The toolbox features an ever-growing set of model components available for the users, with extensive documentation. Components are designed to be interchangeable, such that conceptually related components take the same input and return the same output format and shape. Overall, the general framework requires only a relatively small amount of code to be written by the user. These components constitute the core modules of cpm.models and cpm.generators. At an implementation level, they provide essential components such as learning rules and decision policies; parameter objects that define names, bounds, transformations, and defaults for free parameters and initial states; and wrappers that turn model specifications into executable objects for simulation and estimation. This modularity makes models efficient to construct, compare, reuse, and extend. As far as possible, these components encourage implementations that process the same trial representations and expose standardized interfaces to cpm.optimisation for downstream parameter estimation.

That is why the entry-point of *cpm* is the model specification, because the rest of the workflow depends on having a model that can process trial-level data in a standardised way. Ready-built applications in cpm.applications are the end-product of this process.

Most models in the toolbox are implemented as a state-list processor [11,38], which is most commonly employed in models of learning and decision-making. The state-list processor schema starts by taking in the model parameters and the initial state of the model (e.g., starting Q-values). It then processes the trials presented in the order in which they appear in the data, after each of which the models update their internal states. Notice that, while the model specification relies on user-written code, the internal updates and data organisation rely on toolbox functionalities implemented in the cpm.generators.Wrapper (see below). The goal of this implementation approach is to facilitate model comparison, such that different models will subscribe to the same data-processing representation as much as possible. In general, the toolbox is agnostic as to what the model of interest is, and it can support a broad range of models capable of making trial-level predictions. General-purpose Wrappers to accommodate user-defined custom models that require global processing of data are also in the works. Beyond state-list processors, the toolbox also includes a ready-to-use implementation of a metacognitive measurement model based on Signal Detection Theory [39], which operates on summary statistics rather than trial-level data.

Models are typically made executable through a Wrapper object, which is the central building block of the software architecture. It encapsulates the model function, the data from a single experimental session, and the parameters (Parameters object). It is built to effortlessly simulate, organise model output, store and export simulation results, reset model states, debug, and explore model behaviour (see section Simulating with cpm for simulating larger datasets with multiple participants). Wrapper objects can also serve as checkpoints, where users can export a model that they built, with its specified loss function, so as to pass it onto third-party software; see the ***Interacting with third-party libraries*** section. The cpm.generators.Wrapper class is built to transform the code users write into a generative model.

#### Parameter management system.

Parameterisation is a key component in building meaningful models, because parameters link the formalism to psychologically interpretable constructs and, in the best case scenario, to underlying cognitive processes. For example, a learning rate parameter reflects how quickly an agent updates beliefs in response to new information [40]. In feed-forward network models, parameters such as attentional shift rate and normalisation describe how selectively or uniformly attention is distributed across stimuli [41–43].

In *cpm* this is done via the cpm.generators.Parameters, a unified container for all model variables. It holds both free parameters and any latent variables (e.g., initial values or weights) that should persist or update across trials. Parameters are defined as keyword arguments and stored as attributes, which are wrapped or specified with the cpm.generators.Value object that carries the current value together with parameter bounds and a prior distribution (e.g., truncated normal). These classes expose utilities to enumerate free parameters, return parameter bounds in the order expected by the optimisers, compute the joint prior density, and sample new parameter values from their priors for stimulation or as starting points for optimisation.

**Fig 2:**
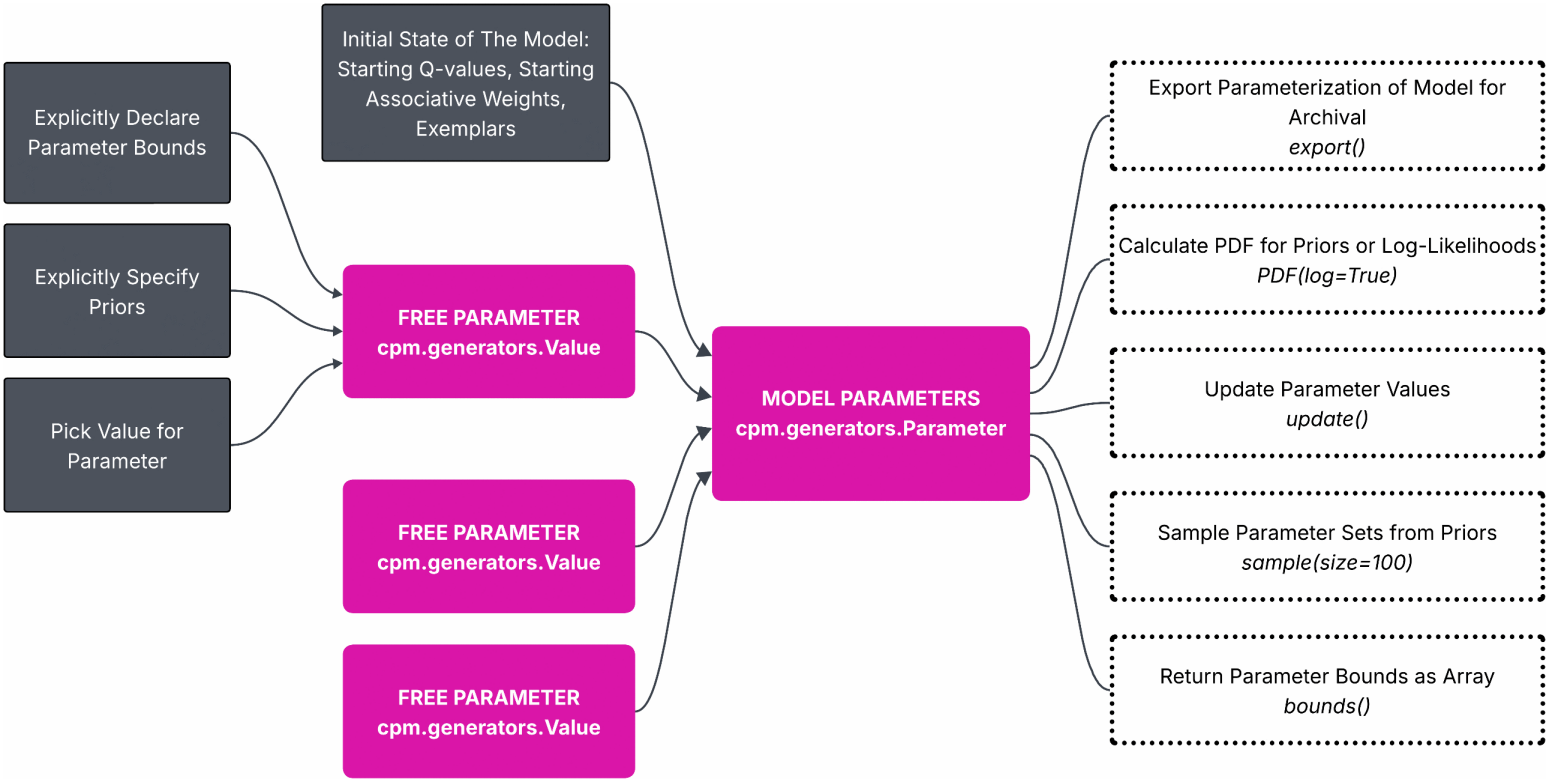
A visual depiction of how parameterisation of a given computational model takes place inside *cpm.* The gray boxes show user-defined inputs to the different functions in the toolbox. Pink boxes show the *cpm* functions wrapping the user-defined properties of the parameter into python objects, and boxes with dotted borders show the functionalities (various python methods) we provide for users. Arrows indicate the input direction, whereas solid lines without arrows indicate the association of the functionalities. In the flowchart above, user-required inputs (in gray) are used to specify either various variables for the model or freely-varying parameters with the cpm.generators.Value class, which is then input to the cpm.generators.Parameters wrapper. Then, the cpm.generators.Parameters supports all functionalities listed in the dotted boxes.

An overview of the essential and expanding set of functions *cpm* provides for model parameters is shown in Fig 2. These methods are often used internally by the library; for example, evaluating the probability density functions (PDFs) of priors enables seamless transition of any model into a hierarchical one without requiring users to hand-code the transition. The parameter management system in the toolbox implements these computations and applies them when requested. This enables users to focus on building, testing, and experimenting with their models, all without the need to reinitialise priors or other parameter attributes.

#### Model execution framework.

The cpm.applications module provides ready-to-use implementations, which are out-of-the-box solutions to fit the most commonly used experimental and analytical scenarios. For example, cpm.applications.decision_making provides a ready-to-use implementation of prospect theory for risky decision-making tasks. These models can also be used as templates that users can tweak and modify for their own use case. Users can directly import and apply these models by specifying the data and relevant problem dimensions. These fully pre-built models provided by the toolbox are often scalable, and come with options to define details of the experiment being fitted upon initialisation (such as how many stimuli are being shown, how many arms or options a participant can choose from on a given trial, etc.) For example, the reinforcement learning model on Listing 2 generalises to experiments with any number of stimuli regardless of how many were shown on each trial. This allows users to reuse code and models across different experiments that vary in complexity. This is the standard approach that all fully pre-built model implementations will attempt to adhere to, within reasonable limits.

Custom models in *cpm* are created by defining a trial-level model function and wrapping it with cpm.generators.Wrapper. The model function must operate with a signature model(parameters, trial, ...), where parameters is a structured cpm.generators.Parameters object; and trial is typically a pandas.Series (or a dictionary) containing the task inputs for that trial. The model function returns a dictionary containing at minimum a dependent entry: the model-generated prediction that will be compared against observed in the loss function (e.g., the probability of the observed choice). The function may also return updated state variables; any returned keys that match parameter names are automatically carried forward to the next trial. This design supports both fitting (using an observed column) and simulation (sampling choices from the model’s policy).

During execution, the model wrapper processes the data trial by trial. The cpm.generators.Wrapper determines the number of trials, and iterates over trials in the order in which they are present in the data. For each trial *t*, the wrapper calls a user-defined model function and saves its output. Critically, it must include a dependent entry (e.g., a choice). Models implemented within the cpm.applications module work via the same mechanisms. This ensures ease of contribution, as completed modelling pipelines with custom models can become valuable additions to the model catalogue.

**Defining generative models:** Generative models take modelling a step further, from simply producing a probability of making an observation to actually producing a discrete response. In practice, turning any model into a generative one is straightforward as long as it already makes trial-level predictions. This conversion is handled within the cpm.generators.Wrapper, but it necessarily requires users to specify the response-generation assumptions that map continuous model predictions to discrete observed actions. A model without being fully generative can be used for fitting, but generative support requires an explicit sampling mechanism. Most often, this mapping involves some form of a likelihood–a decision rule, such as the SoftMax–that can be found in the cpm.models.decision_rule with the corresponding response-generating functions. SoftMax output can then be used as a means of a probability distribution from which one samples a discrete response (e.g., probability of choosing an item over an alternative). These generative models often underlie various pre-data model evaluation frameworks [18] or exploration of global model behaviour [44].

#### Data format and processing.

*cpm* operates on *trial-level* behavioural data, which is most commonly input as a pandas.DataFrame where each row is a trial and each column is a task variable consumed by the model (e.g., stimulus identifiers, outcomes/rewards, condition flags). Each trial must include all task variables required by the model (e.g., stimuli/arms, outcomes, condition flags), and— if fitting is to be performed—an observed variable representing the participant’s response on that trial. A warning is issued if observed is missing, and an error is raised if optimisation is requested without it. For multi-participant datasets, the same tabular data representation can incorporate an identifier column (e.g., participant ID ppt) and be grouped internally to fit participants separately.

Model implementation in cpm.applications typically tolerate flexible column naming by searching for prefixes (e.g., “arm*”, “reward*”), but the essential requirement is that each trial provides all inputs needed by the model and, for optimisation, a well-defined observed target with compatible shape. We provide utility functions in cpm.utils.data for converting experimental data to cpm.application-compatible formats with appropriate column names.

For optimisation, *cpm* converts the wrapper into an objective function via Wrapper.connector(loss, prior). This connector extracts the observed series from the data and returns a parser that maps a candidate parameter vector to a scalar objective value. Internally, the objective resets the model with the proposed parameters, re-runs the full trial loop to generate an array of predicted dependent values, then computes a loss (e.g., negative log-likelihood) by comparing predicted and observed. Optional prior terms can be added by evaluating the parameter prior density and augmenting the objective. *cpm* includes explicit input validation (data shape checks and NaN/Inf diagnostics) to ensure that predicted and observed arrays are compatible and numerically well-defined.

The same data interface supports both fitting and simulation. In fitting mode, the model function typically uses the trial’s observed response to evaluate the likelihood contribution (via dependent). In generation mode, models can instead sample a response from the decision policy (e.g., SoftMax choice or any other likelihood) and return the sampled choice while still emitting dependent outputs; this enables synthetic data generation and parameter-recovery workflows using the identical wrapper and export machinery.

#### Optimisation and inference.

*cpm* features several approaches for estimating the best-fitting parameters of a model. These optimisation procedures are implemented so that they are compatible with the way model building takes place in our framework and that they adhere to our modular approach. The toolbox also assumes that researchers—especially in computational psychiatry—estimate model parameters for individuals, as opposed to estimating a single set of parameters for data pooled across individuals. This approach preserves inter-individual variance and avoids distortions that can arise from aggregate fitting [45,46]. This subject-level estimation approach also underlies various hierarchical estimation methods in the toolbox that can be used to estimate group-level properties of data within the model’s framework in the form of priors. We discuss these approaches below. Therefore, all optimisation routines available in the toolbox automate parameter estimation at the subject level with built-in parallelisation, see the Performance and parallelisation section. The actual algorithms that optimise a given model’s objective function (i.e., “find” the best-fitting parameter values) are imported from established scientific libraries, such as SciPy [47], and are repurposed here to avoid codebase fragmentation that would lead to unnecessary divergence – more informally, we do not wish to “reinvent the wheel” when robust implementations exist.

In line with the scope of *cpm* as a theory-driven modelling toolbox, it does not assume differentiability of likelihood functions. Our inference and optimisation routines are intentionally objective and agnostic. Users pick or provide their custom likelihood and loss functions, and the choice of the optimisation routine should be guided by its properties (e.g., simulation-based or gradient descent).

**Hierarchical estimation:** The *cpm* toolbox supports both regularised non-hierarchical estimation and hierarchical estimation within a unified framework. During the initial model parameterisation, users should define prior distributions for individual parameters. By setting the prior = True flag, these priors can be incorporated into model fitting in two different ways.

First, when treated as fixed, priors act as regularisers during optimisation, such that model fitting proceeds via maximum a posteriori (MAP) estimation. This means that the objective function for optimisation is the log-posterior density, that is, the sum of the log-likelihood (summed across trials) and log-prior density (summed across parameters). In this setting, the hyper-parameters of priors are given by the user and remain fixed during fitting.

Second, *cpm* provides hierarchical estimation routines in which the hyper-parameters of priors are estimated from the data. Importantly, users are not required to explicitly define group-level models; instead, hierarchical structure is induced implicitly by treating the priors on individual parameters as group-level distributions whose parameters are iteratively updated. This design lowers the barrier to hierarchical modelling while retaining flexibility in model specification.

**Fig 3:**
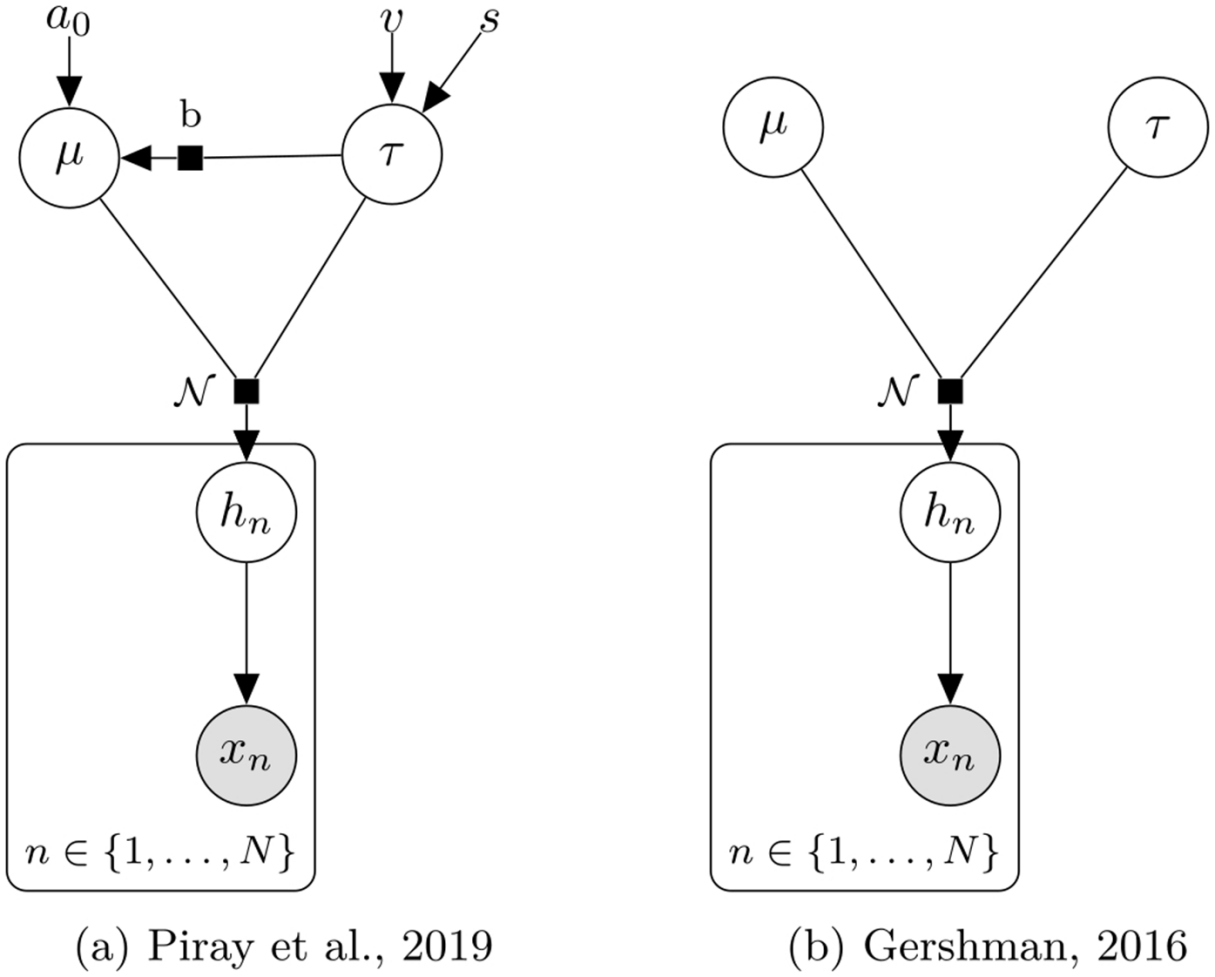
Comparison of two types of hierarchical estimation methods supported by *cpm.* Panel **(a)** illustrates the model constructed by the toolbox for variational Bayesian estimation [48]. Panel **(b)** illustrates the model constructed by the toolbox for empirical Bayesian estimation [49]. These are simplified schematic depiction of the models using plate notation [50]: shaded nodes represent observed variables (i.e., data) whereas white nodes represent latent variables (i.e., model parameters); directed edges (i.e., arrows) indicate dependencies between variables; “plates” are used to group variables that repeat together; and variables that are not enclosed by a circle represent fixed values (i.e., hyper-parameters). Variable names and annotations as follows. *n*: participant index; *N*: total number of participants; $x_{n}$: participant-level observed data; $h_{n}$: participant-level parameter; 𝒩: normal distribution; μ: group-level parameter mean; τ: group-level parameter precision (inverse variance); *a*_0_, *b*, *v*, and *s*: scalar values that define the priors on μ and τ (see [48] for details).

Currently, *cpm* implements two approaches for estimating prior hyper-parameters: an empirical Bayes procedure [49] (see Fig 3B) and a variational Bayes method [48] (see Fig 3A). Note that the variational Bayes approach as introduced in [48] is implemented only partially in *cpm*, focusing on estimation of hyper-parameters for priors, while removing the parts of the procedure that enable multi-model comparisons. This decision was driven by two factors: (i) methods related to model comparisons are organised by different sub-modules; and (ii) we are yet to validate the model comparison components of the original implementation in [48] internally. Both approaches follow an expectation-maximisation scheme [51], iteratively alternating between estimating participant-level parameters (given current priors) and updating prior hyper-parameters (given current participant-level parameter estimates). In the empirical Bayes approach, group-level means and variances of parameters emerge as point estimates from this iterative procedure, rather than being treated as explicit latent variables. The variational Bayes approach, in contrast, maintains approximate posterior distributions over parameters (including their group-level means and variances), thereby providing a richer representation of estimation uncertainty. These two methods are implemented in the cpm.hierarchical module as EmpiricalBayes and VariationalBayes, respectively. Their input arguments match, and they can respond to the same method calls due to the modular design of *cpm*.

These hierarchical methods improve robustness to noise and outliers, and help constrain extreme or implausible parameter estimates [46,52]. However, their performance depends on the choice of prior distributions. The selection of priors is non-trivial and will affect how likely model predictions and model parameters become: narrow priors can dominate the likelihood and bias estimates, while broad priors may fail to regularise individual estimates in an effective way. As a practical starting point, we recommend “weakly informative” priors centred on plausible parameter ranges, which can be refined using domain knowledge or previous empirical findings (see also [53] for guidance). The toolbox supports all distribution functions implemented in SciPy, but the cpm.generators.Value class allows custom-written distribution functions to be supplied as long as their method arguments match those of the SciPy distribution functions.

The current implementation focuses on optimisation-based hierarchical methods (MAP, empirical Bayes, and variational Bayes), and does not include fully Bayesian sampling-based approaches. This reflects a design choice, prioritising computational efficiency and ease of use. However, the modular structure of *cpm* allows for additional inference methods to be incorporated in future extensions.

**Goodness-of-fit:** The toolbox already implements several goodness-of-fit metrics. These include standard likelihood-based and various non-parametric measures (e.g., $\chi^{2}$, sum of squared errors) of model performance. Line 10–24 in the Listing 6 shows how users can define an optimisation routine. User-defined functions can also be used, as long as the input arguments correspond to those implemented in cpm.optimisiation.minimisation and the function is callable. A callable function is any function that you can call by using a pair of parentheses and (optionally) a series of arguments.

**Interacting with third-party libraries:** The Wrapper class allows users to export an objective function that is compatible with almost all fitting procedures available in other libraries. Most optimisation methods only require a callable function that takes in some parameters and returns a goodness-of-fit metric. It can be done through objective = generative.connector(minimisation = minimise.LogLikelihood.bernoulli), where the resulting objective function is a Python function object and can be passed on to any third-party method for optimisation.

#### Performance and parallelisation.

The toolbox is optimised for typical behavioural modelling workflows and scales via parallel execution when fitting many participants. The core execution model is a lightweight trial loop in Wrapper.run(), where model operations performed sequentially; updates are carried out in-place via the Parameters object, reducing runtime for recalculations via a single pass forward through the data (e.g., there is no need to access and manipulate large DataFrame objects). For parallelisation, the toolbox supports distributing work across CPU cores–primarily at the *participant level*– using ipyparallel (a parallelisation backend for Jupyter environments) and multiprocessing-style backends in the optimisation and application layers. Several application models explicitly declare worker-side dependencies (e.g., @ipp.require("numpy")) to ensure remote engines can execute the model function, and utilities exist to detect available cores and manage parallel execution.

#### Testing and stability.

To ensure correctness and stability, *cpm* is accompanied by an extensive suite of automated unit tests implemented with pytest. These tests cover deterministic numerical components (e.g., learning rules), objective functions used during fitting, and ready-to-use model implementations. They also include checks for edge cases (e.g., NaN/Inf inputs, shape mismatches, etc.) to ensure robust performance under a wide range of conditions.

In addition, regression tests continuously verify that model predictions, optimisation outcomes, and archived results remain stable across code changes and dependency updates. This promotes reproducibility and reliable reuse of existing workflows, and prevents unintended breakage when the codebase changes (e.g., when new features and models are added or existing components are refactored). As such, regression tests provide guardrails for third-party contributions by ensuring extensions do not silently break existing behaviour or invalidate past simulations. All tests are automatically run on each version update.

### Related Work

A growing ecosystem of open-source toolboxes support computational modelling in cognitive science, serving a broad audience that includes computational psychiatry. These toolboxes can be broadly grouped according to their primary focus: (i) specific model families, (ii) curated sets of tasks and theoretical frameworks, and (iii) inference methodologies.

The first category of toolboxes focuses on specific model families. One prominent example is evidence accumulation models, especially the diffusion decision model (DDM) [54,55]. These models have become a central theoretical framework in cognitive psychology [56] and neuroscience [57], and are increasingly used to characterise latent mechanisms underlying neuropsychiatric symptoms [58]. Accordingly, there is a strong history of mature software packages implementing these models [59–63]. The Python library HSSM [64] (building on its widely-used predecessor HDDM [65]) and the R package EMC2 [66] both provide a state-of-the-art Bayesian workflow [67] for specifying, estimating, and critically evaluating several variants of evidence accumulation models. These packages also cover elements of model construction, using formula syntax analogous to linear mixed-effects modelling [68] to flexibly express experimental designs. HSSM was also explicitly designed to foster community contributions around model implementations. Another Python library, PyDDM [69] emphasises flexible model specification, enabling complex decision dynamics such as leaky integration and time-varying response thresholds. Taken together, these toolboxes provide powerful and well-developed environments for implementing evidence accumulation models, and *cpm* does not aim to replicate this level of advanced coverage.

Another prominent example of toolboxes focused on a particular model family is Bayesian models of learning under uncertainty, especially the hierarchical Gaussian filter (HGF) [70,71], which has been widely applied to explain aberrant belief updating in psychiatric populations, including in schizophrenia, autism, and affective disorders [72–74]. The HGF has been implemented in several well-developed toolboxes [75,76]. As with evidence accumulation models, *cpm* does not aim to duplicate this coverage, instead aiming to provide a unified modelling workflow that can be applied across multiple theory-driven modelling traditions used in computational psychiatry.

A second category of toolboxes focuses on specific task paradigms and associated theoretical frameworks. These include, for example, hBayesDM [77], which performs hierarchical Bayesian estimation for a curated set of models of widely-used decision-making tasks; catlearn [11,38], which focuses on implementing established learning and categorisation theories; the MemToolbox [78] for mixture models of visual working memory tasks; and the TreeBUGS package for multinomial processing tree models of recognition memory [79]. These approaches are highly effective when the user’s task and model fall within the supported scope. By design, *cpm* targets a broader set of modelling approaches (across tasks, model families, and inference routines), with an explicit focus on standardised end-to-end workflows and flexible model building; and it does not restrict users to select from a set of pre-defined models.

A third category of toolboxes focuses on inference methods. Two examples include BayesFlow [80–82] and sbi [83], which are both built as simulation-based inference frameworks. Unlike the previous approaches, these are organised around the inference method and ask users to supply their own simulator and perform likelihood-free posterior estimation, typically via training neural networks. This vastly expands the space of tractable models, since any generative simulator can be fitted regardless of likelihood tractability. For example, this approach has proven fruitful for estimating complex extensions of evidence accumulation models [84,85]. The scope of these toolboxes is, however, deliberately narrow with respect to model construction; the simulator itself is the user’s responsibility. *cpm* is complementary rather than competing: it provides the model construction and workflow, and in principle simulators built within our toolbox could be paired with either sbi or BayesFlow for inference in settings where likelihoods are unavailable.

Lastly, an emerging line of work departs from explicitly specified cognitive models by training recurrent neural networks directly on behavioural data to infer latent task dynamics in a data-driven manner [86]. When combined with sparse equation discovery methods [87], these approaches can yield interpretable dynamical systems that give new insights into cognitive mechanisms [88,89]. While promising, these tools are conceptually distinct from theory-driven modelling approaches and often require substantial expertise in machine learning.

Taken together, *cpm* is positioned as a Python framework intended to harmonise model construction and estimation across a broader set of computational psychiatry problems and models.

## Results

In what follows, we provide a walk-through of the modelling pipeline to showcase features of the toolbox covering all aspects of our minimal modelling workflow in Fig 1, from specifying parameters to estimating group-level parameters, all within the framework of *cpm*. At every stage, the online documentation covers function usage with examples and references to relevant methodological literature. Installation instructions and examples are available as described in the Availability and future directions section. The code included in this paper is available a single Jupyter Notebook file on Google Colab.

Starting off the coding journey, *cpm* can be imported just like any other Python library. Listing 1 shows a straightforward way to import the complete library with all available sub-modules. Later code snippets in the text will demonstrate how to important sub-modules or particular functions.

**Listing 1:**
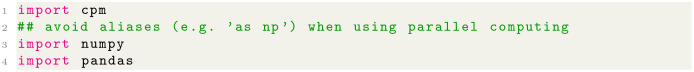
Importing the cpm toolbox and its dependencies in a Python environment.

Before we move on to the walk-through, in which we demonstrate the toolbox through concrete examples, we briefly introduce the multi-armed bandit task that will serve as an example throughout the demonstration. Bandit tasks are widely used and familiar to many researchers in the general domain of cognitive science, making them a suitable example. Please note that the toolbox can handle many other tasks and data structures. The current example has been chosen to demonstrate core functionality in an accessible way.

In a canonical bandit task, participants repeatedly choose between alternatives, often referred to as arms, to earn rewards, with the overall goal of maximising the cumulative rewards earned by the end of the experiment. Each option is associated with an underlying probability of generating a reward. For example, if the stimulus has a corresponding reward rate of 80%, it–on average–results in a reward on 80% of the trials and in no reward on 20% of trials. In our current experiment, each trial presents two stimuli (arms) out of a battery of four, participants select one, and then receive binary feedback indicating reward or no reward.

Table 2 shows the data based on a two-armed bandit task. Each row is a trial (or state of the environment) and columns contain information about that trial. The data include the participant identifier, *ppt*; the trial number, trial; the stimuli appearing on left and right side of the screen (*arm_left* and *arm_right*); the reward for each option, *reward_left* and *reward_right*; the *response* of the participant, where 0 is left and 1 is right; and the *feedback* the participant received after selecting either left or right. The four possible stimuli are denoted by integers 1–4, out of which two appears on each trial. If stimulus 1 appeared on the left and stimulus 4 appeared on the right, then *arm_left* will equal to 1 and *arm_right* equal to 4 (as in the final row of Table 2).

**Table 2:** First five rows of one of the datasets included in the cpm toolbox. The data is based on a two-armed bandit task for one participant with a total of 4 unique stimuli from which only two can be chosen among on any given each trial.

ppt	trial	arm_left	arm_right	reward_left	reward_right	response	feedback	observed
1	1	2	4	1	1	0	1	0
1	2	2	4	1	1	1	1	1
1	3	2	1	0	0	0	0	0
1	4	2	1	0	0	1	0	0
1	5	1	4	0	0	0	0	0

### Constructing the model

In this walk-through, we focus on a learning model and implement it as a stateful list processor. The complete mathematical treatment of the model can be found in S1 Text and a pseudocode version in S1 Algorithm. Here, we demonstrate two ways to specify such models within *cpm*: either using pre-existing, ready-to-use implementations, or building the model from scratch.

#### Using existing models via *cpm.applications.*

cpm.applications offers a curated catalogue of ready-to-use implementations for the most commonly used models in the field. In Listing 2, the *RLRW* (Reinforcement Learning with the Rescorla-Wagner learning rule; Wrapper) class is initialised with the data of a single participant; the dimensions = 4 relates to the four possible stimuli. The model is initialised with a predefined set of default parameters, but the user has the option to specify them; see the function documentation. Note that running this code will produce a warning, as no parameters are explicitly specified. All fully built model implementations are available in cpm.applications and belong to the Wrapper class (see Making models executable). This means that everything that can be done with the Wrapper can also be done with the built-in applications. The model.run() command runs the model on the data and calculates the predictions of the model, while model.export() organises the results into a pandas DataFrame.

**Listing 2:**
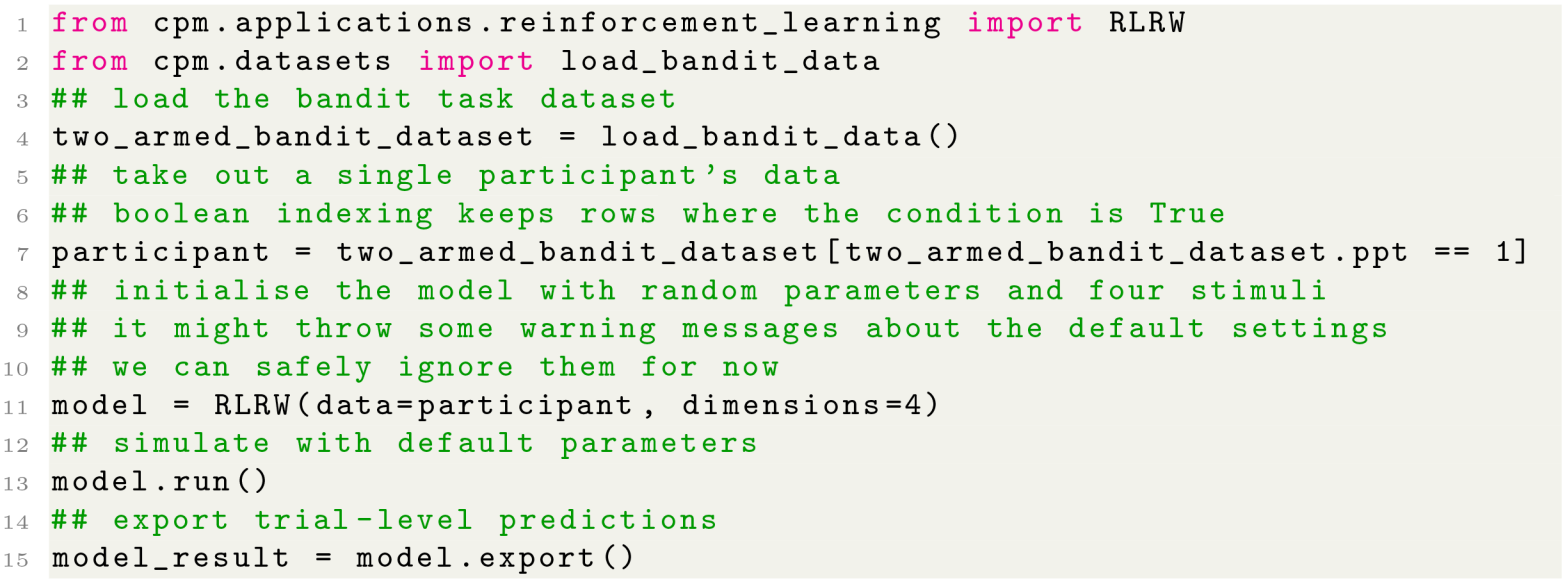
An example of a built-in reinforcement learning model using a version of the delta learning rule with the SoftMax decision rule suited for k-arm bandit tasks.

Note that the *RLRW* wrapper implements a single variant of a class of reinforcement learning models using the delta rule [36,40,90] to update *Q*-values and an exponential ratio rule (SoftMax [37,40]), to turn *Q*-values into choice probabilities (policy). We can imagine dozens of variations to this single model, such as one using a choice rule different from the exponential choice rule (or ratio rule). Users can implement such variants by copying the open source code from the public repository of the toolbox and replacing the cpm.models.decision.Softmax module with alternatives, such as cpm.models.decision.GreedyRule, or altering it to their needs.

#### Building a model from scratch.

**Managing parameters with** **cpm.generators.parameters:** In the previous example, we relied on the toolbox for default parameters by using built-in applications. In many cases, we want to define our own priors and parameter bounds, or tweak specific model components (e.g., the learning rule). We may also want to create our own template model that we can modify and expand as our analysis evolves. Listing 3 therefore defines parameterisation for a custom model closely mimicking the one from the earlier example, and Listing 4 shows the corresponding model implementation. Archival is further supported by the cpm.generators.Parameters.export() which documents bounds and priors by exporting the parameterisation to a pandas.DataFrame.

**Listing 3:**
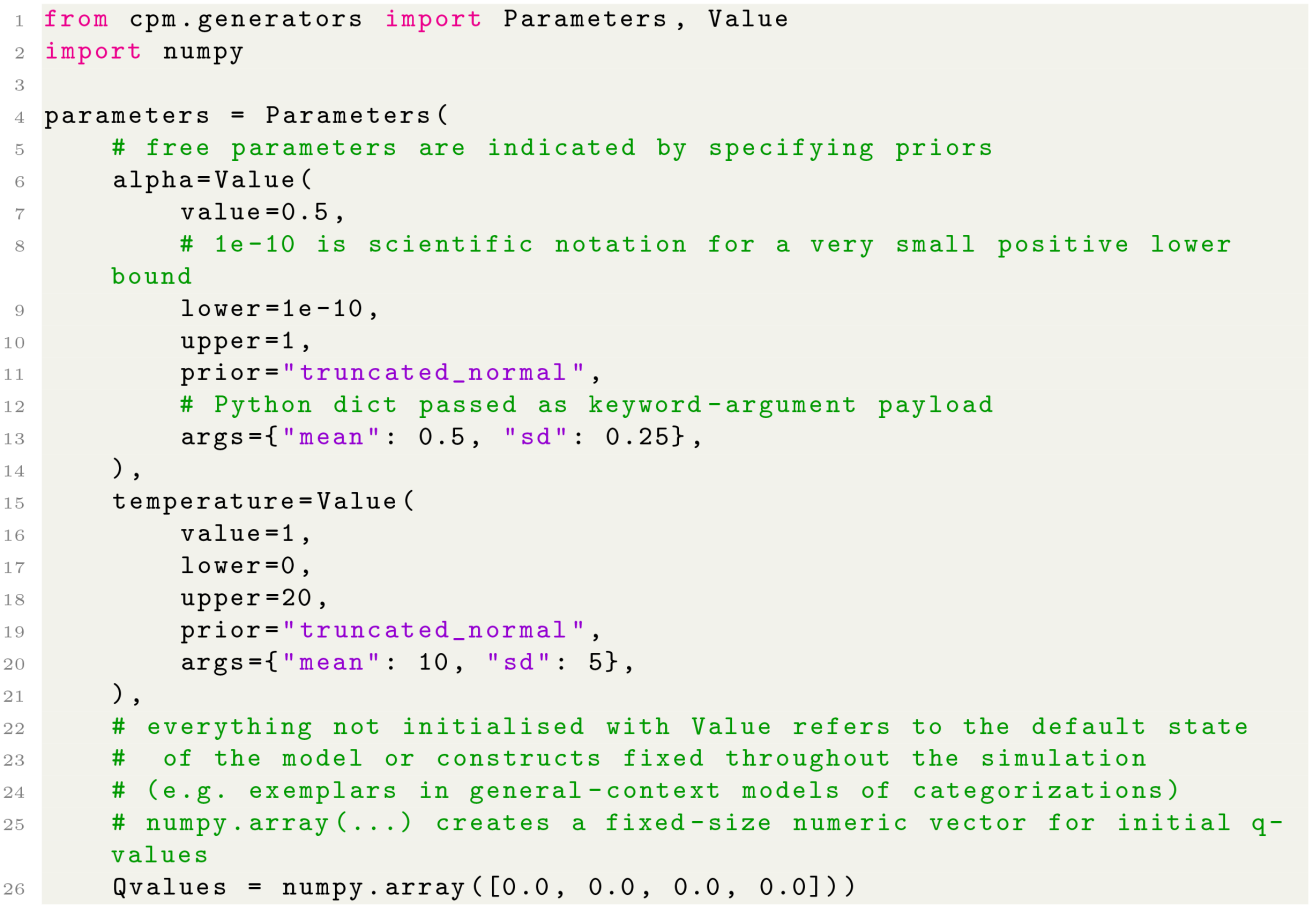
“Specifying parameters in cpm”.

**Listing 4:**
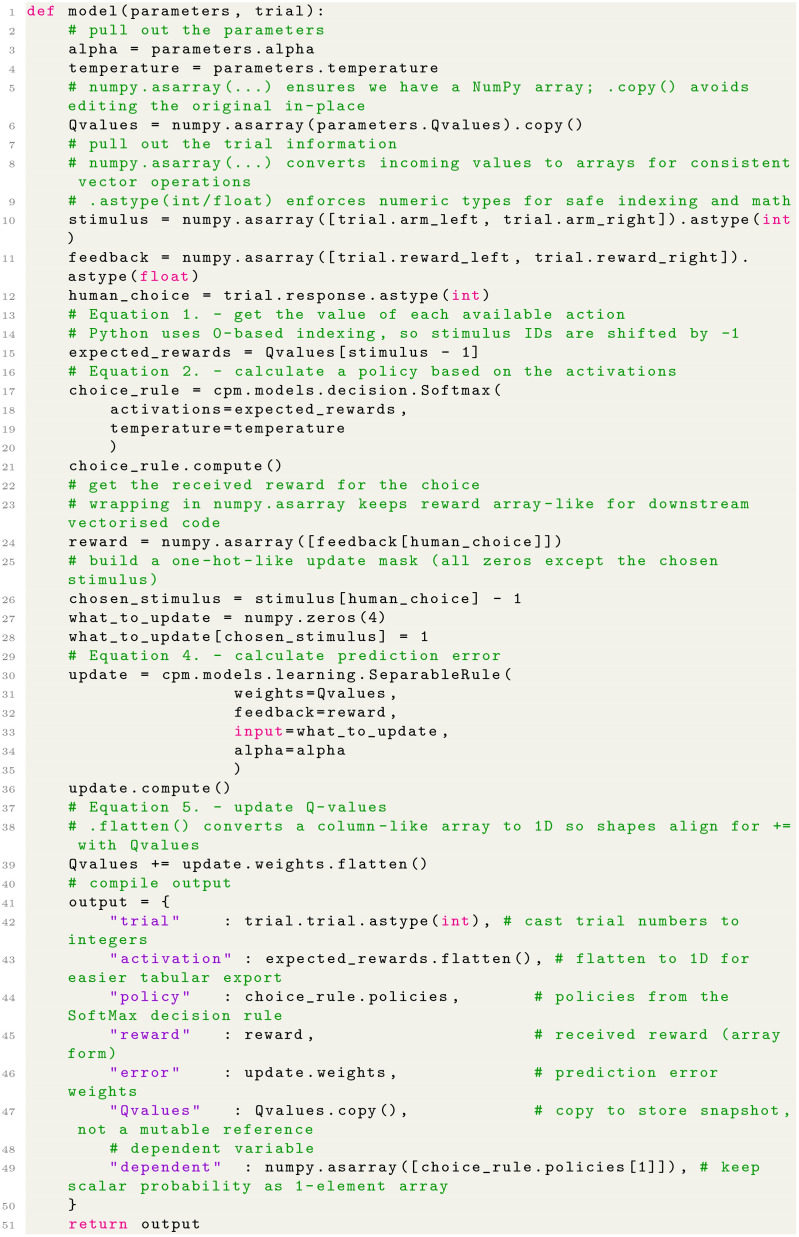
An example delta rule reinforcement learning model implemented in cpm. Equation numbers correspond to the ones found in the S1 Text.

We begin by specifying two free parameters: *alpha* for the learning rate, and *temperature* parameter governing the choice stochasticity. These two parameters have accompanying lower and upper bounds (i.e., minimum and maximum values these parameters can take), and group-level parameter constraints in the form of priors with various hyperparameters (e.g., distribution types, means and standard deviations). Users can specify these by using the cpm.generators.Value class shown on Listing 3. Users must configure the settings for every parameter. Anything else, such as parameters that are fixed between participants or derived variables reflecting some latent state of the model (i.e., quantities that do not need to be estimated), can be specified without the cpm.generators.Value class. Examples include *Q*-values in reinforcement learning models [40], which encode the “rewardingness” of a stimulus, or transition matrices in model-based and model-free learning [30], encoding the likelihood of different stimuli following one another in multi-step decision-making.

**Specifying processes with** **cpm.models:** After defining custom parameters, users write a Python function taking in the cpm.generators.Parameters object we defined above and the current trial (one row in the dataset) as a pandas.Series; more information on this class can be found on the official *pandas* documentation. This function must define operations in a fixed sequence: (1) extracting parameters; (2) extracting latent variables of the model; (3) define the internal model operations (e.g., decision or learning rules for predictive learning models); (4) define the function output to track latent variables; (5) define the dependent variable of interest that will be compared against empirical data (e.g., choice probability of the current action).

Listing 4 shows this specification in *cpm*. Lines 3–13 of Listing 4 extract parameters and latent variables, and Lines 41–50 specify the model output, which includes the dependent variables on Line 49. Everything in-between relates to the computations that take place on a given trial in our two-armed bandit experiment. Here, we decided to use a SoftMax [37] and a variant of the delta rule [36, 40, 90], respectively, present in lines 17–21 and lines 30–36.

Listing 4 uses two of our “building blocks”: cpm.models.learning.SeparableRule and cpm.models.decision.Softmax, a learning and decision rule, respectively. Each model component has accompanying documentation with appropriate references and usage manuals. Furthermore, each class also includes other variants of the same equations. For example, assume that we intend to try a noisy decision function [31], which increases the probability of selecting options with exceptionally low *Q*-values. Although it may seem counter-intuitive, this helps capture surprising responses due to lapses of attention. In this case, the generic cpm.models.decision.Softmax class includes this version as an associated method, so we just exchange choice_rule.compute() on lines 24 to choice_rule.irreducible_noise(). This allows users to explore how thematically related variants of the same computation impact model fits and behaviours, which naturally promotes model comparison. In terms of general software architecture, this approach also lets *cpm* organise these modifications as part of the same class for ease-of-use and theoretical alignment. Information on what is implemented can be found in the online documentation.

As a final step, each model function must return some output, specifically a Python dict object, specified by the user. Everything in the output will be saved from the trial and organised as a pandas.DataFrame. The single mandatory field that every model specification must output is the “dependent variable”. Additionally, for ease-of-use, if variable names correspond to any entry in the parameter object, such as *Q*-values in our examples in Listings 4 and 3, they will be updated in the parameters object. This small feature helps keep code clean and ensures safe organisation of important variables that must be tracked or iteratively updated for the model. Once the model function has been specified, the toolbox encapsulates the model with the Wrapper class command, thereby simplifying and streamlining the remaining steps of a computational modelling pipeline.

### Making models executable

**Fig 4:**
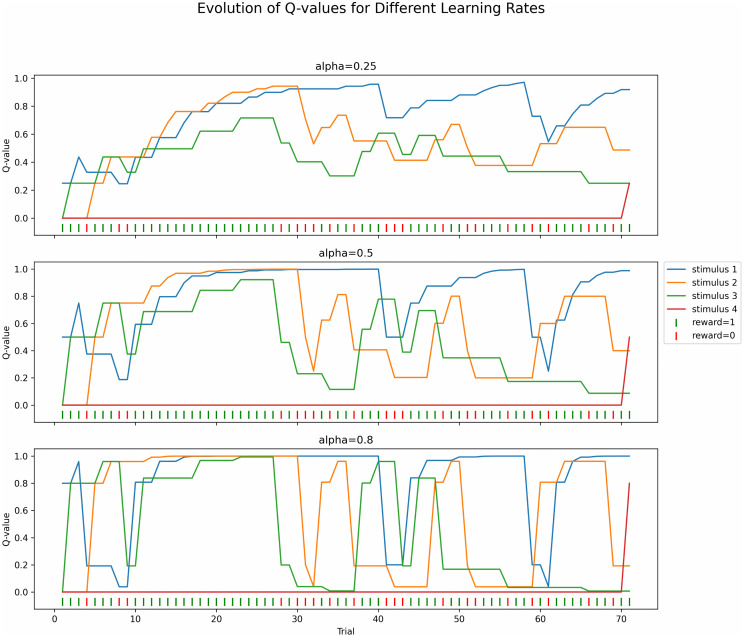
A simulation, with *Q*-values for a given stimulus (colour) on the y-axis and the trial on the x-axis. Ticks below the lines indicate whether the model received a reward (green) or not (red) for its choice on that particular trial. The figure shows three example simulations with three different learning rates, alpha.

Once the parameters and user-written model function are specified, the immediate next steps are usually practical: does the model behave sensibly, do the trial-by-trial calculations match what we think the model should be doing, and can we apply it to the data to inspect the learning curves? In *cpm*, these tasks are supported through the Wrapper, which packages the model and the parameters behind a consistent interface and makes it executable within the workflow. Ready-to-use implementations in cpm.applications are already packaged as Wrapper classes. Once “wrapped”, models integrate cleanly with the rest of the toolbox, enabling simulation under fixed parameters, before moving on to estimating parameters from data, exploring hierarchical estimation workflows, or investigating parameter spaces to understand model behaviour. Listing 5 shows how to initialise this class. The class also supports variety of methods, most notably, it supports the export of all tracked latent variables and other model predictions into a pandas.DataFrame. These exported and organised outputs can be archived or used for analysis of how model behaviour evolves by using different parameters. Fig 4 shows a simulation with different learning rates (alpha) orchestrated by using the Wrapper on the current dataset, taken from one of our online examples (see online documentation).

**Listing 5:**
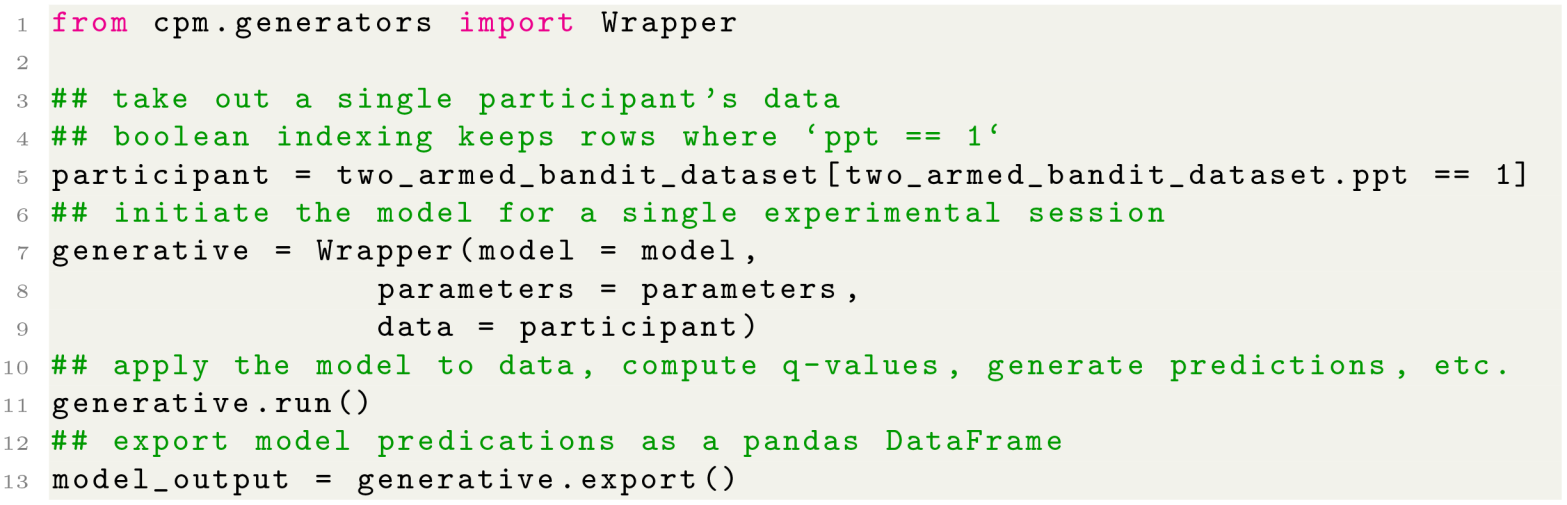
An example code for declaring a Wrapper object using objects from previous Listings.

### Fitting behavioural data

Once the model is finalised, and its trial-by-trial computations are checked, the next step is usually to estimate its free parameters that best approximate the observed responses — i.e., to fit the model to data. Listing 6 presents an example use case. Note that *cpm* takes care of both the parameter estimation for each participant and the parallelisation of the estimations of all participants on multiple CPU cores. The toolbox facilitates this process without requiring scientists to have detailed knowledge of the various methods of implementing parallelisation in Python or within Jupyter kernels.

**Listing 6:**
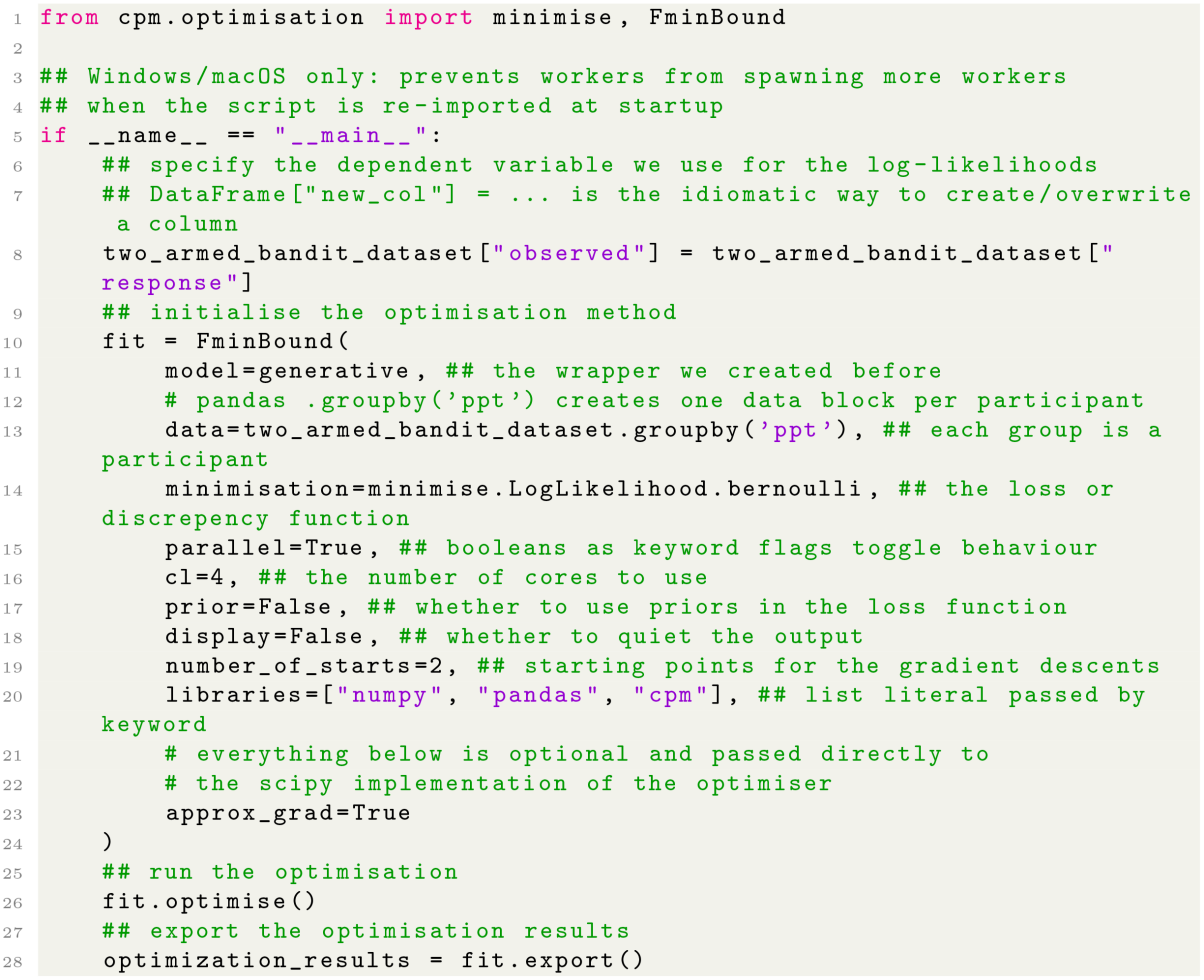
An example code for optimising parameters of a given model with a bounded fmin optimisation method, on a subject-level, with parallel computing. FminBound is a wrapper function that uses a *scipy* method to estimate parameters on a subject-level and organises the data into a tabular format. On Windows and macOS (Python ≥ 3.8), code triggering parallel execution must be wrapped in if _name_ == "_main_: to prevent workers from spawning more workers when the script is re-imported at startup. This requires running the code as a standalone Python script (e.g., python script.py) rather than line-by-line in an interactive session or notebook.

**Fig 5:**
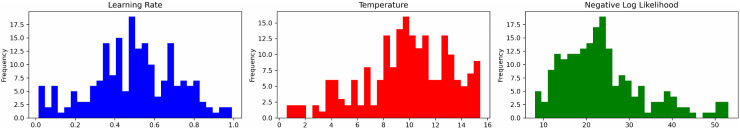
Distributions of estimates of the learning rate (left) and temperature (middle) parameters, as well as the participant-wise summed negative log likelihoods after fitting the model to data with FminBound. See the Examples in the online documentation for the corresponding code.

The estimated parameters are usually not the end-point of the analysis. Researchers often use them to quantify individual differences, perform computational phenotyping, relate latent mechanisms to psychiatric symptoms, compare groups, or use model-derived measures (e.g., prediction errors) as trial-wise regressors. With this in mind, the toolbox efficiently organises model-fitting results into structured pandas.DataFrames, to streamline the further analysis of behavioural data after parameter estimation. Fig 5 shows the parameter estimates from our previous model plotted directly from the DataFrame.

**Model comparison:** Sometimes, goodness-of-fit is not a sufficient criterion to select among competing models, because model selection methods should be sensitive to both fit and complexity [91]. We are actively developing these methods to work in conjunction with optimisation routines to provide ways to penalise models by averaging their goodness-of-fit over the parameter space. See the Future directions section for more information on what other model selection metrics are planned for inclusion. As an interim step, *cpm* provides various model comparison metrics as stand-alone formulas, such as Bayesian Information Criterion (BIC) [92] and Akaike information criterion (AIC) [93]. They are available within cpm.optimisation.compare.

### Hierarchical models with cpm.hierarchical

In computational psychiatry, individual data can be noisy and clinical datasets are often small due to practical constraints on recruitment and testing time. Hierarchical priors address this by stabilising parameter estimates, which yields more reliable individual estimates [46, 52]. This can improve individual estimates for future analysis, such as group comparisons. Furthermore, informative priors will constrain the complexity of the models by placing probabilities over parameters that control for model flexibility by penalising parameters further away from the most likely parameter value [49]. As a principled practice, we recommend using priors and using hierarchical methods when appropriate, as they improve both stability and interpretability of parameter estimates.

**Fig 6:**
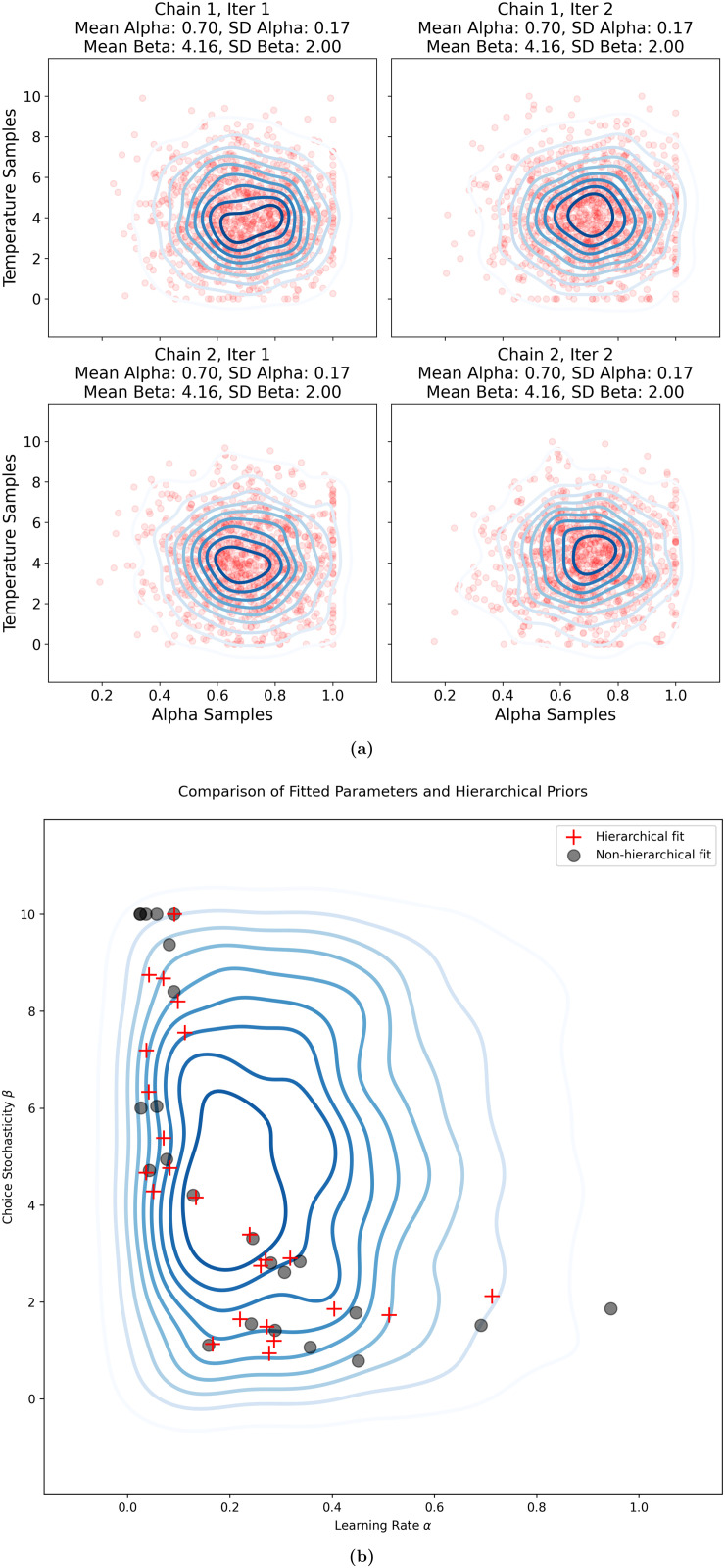
Fig (a) shows the prior landscape for various hyperparameters estimated with the Variational Bayesian Inference available in *cpm*. The density contours (blue lines) plot the distribution of the priors. Darker lines indicate higher density. The red dots are samples from the prior. Each subplot is an estimate from a given iteration in the Expectation-Maximisation algorithm. On top of the sub-figures, we show the means and standard deviations for each parameters. Fig (b) shows a comparison between estimating parameters for a hierarchical and non-hierarchical model with two parameters within the same dataset using the cpm.hierarchical modules. The gray dots are non-hierarchical estimates, whereas the red crosses are the hierarchical model estimates. The density contours (blue lines) plot the distribution of the priors. Darker lines indicate higher density.

In cpm, this is done via setting the prior argument to True within the optimisation routine. This will apply the priors we specified in Listing 3 to the fitting. But it is often not that straightforward. Priors have not traditionally been provided alongside these models, but as their advantage became more apparent in recent years [49,53], their inclusion became more prevalent. In *cpm*, we implemented two hierarchical estimation techniques that find hyperparameters for normally distributed priors that constrain model complexity, while searching for the best fit of the model across all participants. Listing 7 demonstrates how this is carried out within the toolbox. Once the fitting object has been initialised with the prior set to True (Lines 7–21), it can be input to a hierarchical estimation procedure (Line 23–31), which then can carry out the estimation. These methods come with built-in visual convergence diagnostics (Line 37–40) for reviewing the performance of the estimator. Fig 6a shows how the priors changed throughout different iterations of the estimation process, whereas (b) shows how priors “pull” extreme parameter estimates towards the group means. Note that the hierarchical estimates spread much less than non-hierarchical estimates, which shows how extreme parameter estimates are controlled via priors.

**Listing 7:**
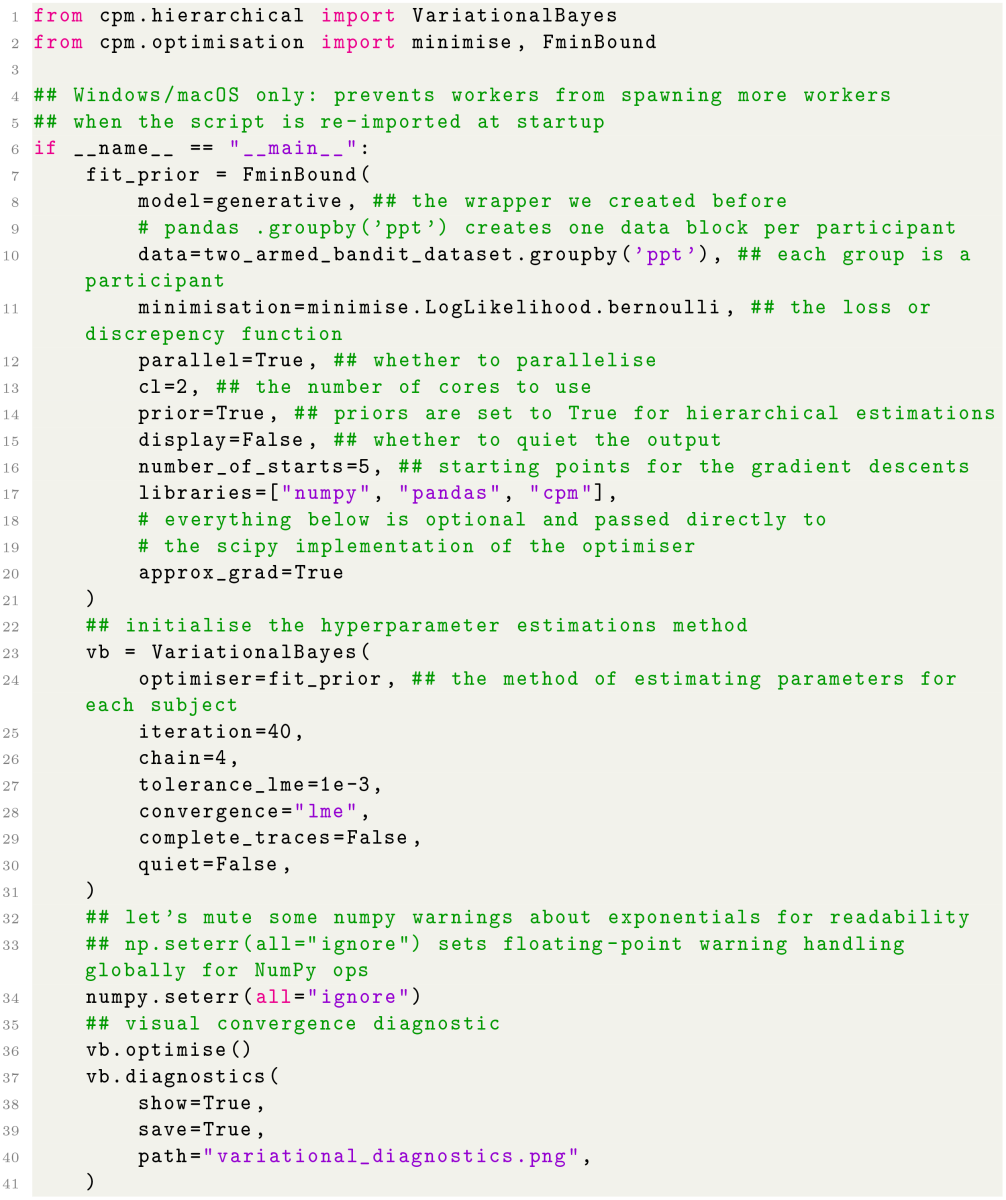
Setting up and running hieararchical estimation within the toolbox. Here, we declare the same optimisation method as before, but set the prior to True, effectively turning the model into a hierarchical one. This object is then passed on to the Variational Bayesian estimation method, where we can estimate hyperparameters.

This type of hyperparameter estimation can map really well to problems in computational psychiatry. One possible application of this hierarchical approach could be in the comparison of clinical groups to healthy controls, where group-level hyperparameters for priors are estimated within each group [94,95], and can provide robust weighting of individual differences as expressed through parameter variation between subjects while simultaneously constraining these estimates. Users could also select priors from previous literature and apply it during the fitting of specific experimental and control groups.

#### Simulating with *cpm.*

**Fig 7:**
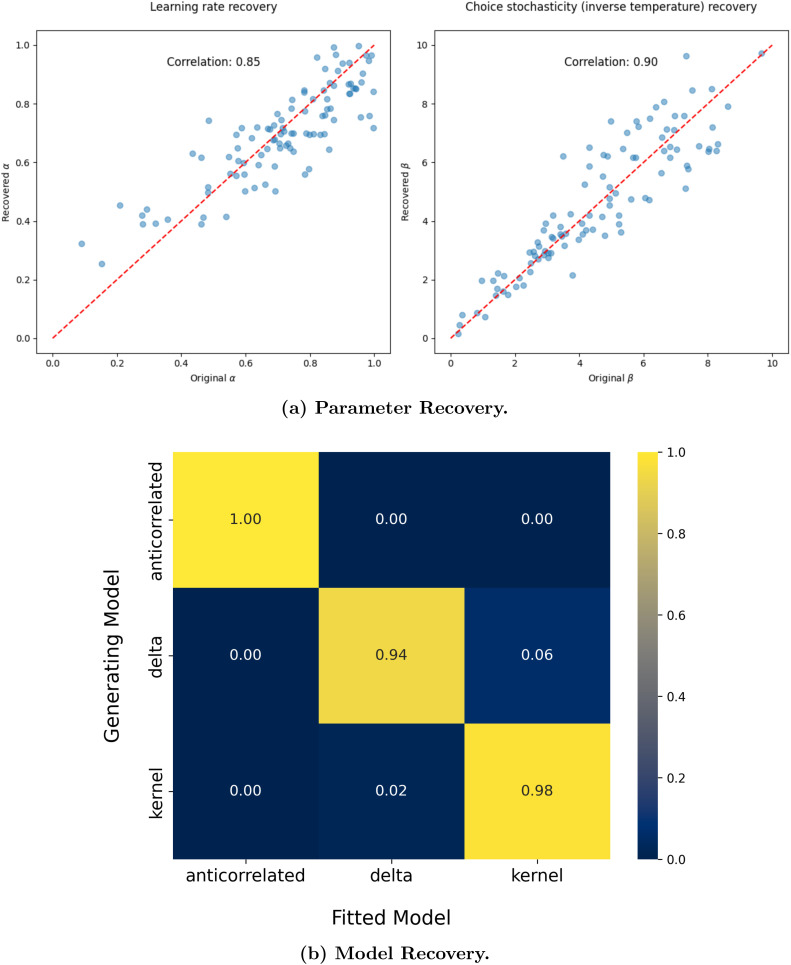
Figures showing pre-data model evaluations for various models. Fig **(a)** shows the parameter recovery of cpm.applications.RLRW in a hierarchical modelling framework. Each dot is a dataset generated by a randomly picked α (learning rate) and β (choice stochasticity or inverse temperature). The x-axis shows the original parameters, and the y-axis shows the recovered parameters after fitting the model to the generated data. Fig **(b)** shows model recovery for three example model: an anti-correlated model with a modified delta learning rule [96], a kernel choice model [18], and a reinforcement learning model with a variant of the delta rule [40]. The numbers are proportions of times the model won over the alternatives out of 100 simulated datasets. For the differences between models, see S1 Text or S2 Algorithm, S3 Algorithm, and S1 Algorithm for pseudocodes, respectively.

After fitting, simulations are usually the next step as they allow researchers to answer practical questions that arise in applied work: Does the model reproduce the qualitative patterns in the data? Is the trial-by-trial behaviour of the model within expectations? Are the estimated parameters actually identifiable via parameter recovery? Simulations also underpin several validation techniques in computational modelling, such as parameter recovery and model recovery. Beyond validation, simulation outputs are frequently used as model-derived regressors in subsequent analyses — for example, relating trial-wise prediction errors to neural activations or reaction times. As such, simulations bridge model fitting, model validation, and later statistical analysis. The toolbox provides methods for simulating with best-fitting parameters and generating data for recovery studies, both of which are done with the object cpm.generators.Simulator. The *Simulator* class streamlines simulations with large sample sizes, and helps with the application of your models to many participants, including managing the resulting data for downstream analysis (e.g., model-based functional MRI analysis) or model validation. Listing 8 demonstrates the standard commands for simulating with the previously estimated best-fitting parameters (Line 4–10). The method runs the model for each participant indexed as each group in the pandas.DataFrame. The Simulator also allows us to generate data for parameter and model recovery, assuming that users defined a generative model (see **Defining generative models** section). This option is available in every complete model implementation, which can then be used as a template to adapt the code for their models. For example, in *RLRW*, we can turn on this option by setting the input argument generate to True. Fig 7A shows the recovery performance of *RLRW* in a hierarchical modelling framework. Data were generated using cpm.generators.Simulator on our example two-arm bandit task and cpm.applications.reinforcement_learning.RLRW was fitted using cpm.optimisation.FminBound and cpm.hierarchical.VariationalBayes.

**Listing 8:**
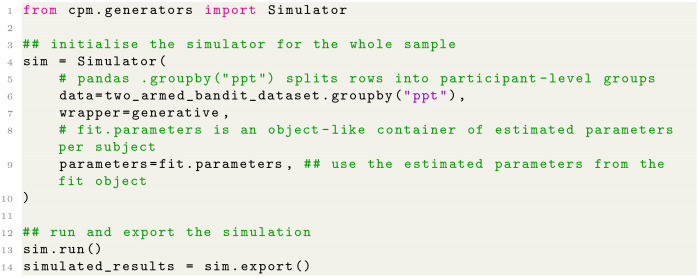
An example Simulator object for simulating with best-fitting parameters. The referenced parameters attribute from Line 10 is part of the cpm.optimisation.FminBound object from our previous fit after running the optimisation on Listing 6.

The entire pipeline –model construction, parameter estimation, hierarchical estimation, simulation– was done within a unified workflow without ever leaving the toolbox, eliminating the need for extensive custom scripting and reducing the risk of implementation error. Combining modules, such as the cpm.generators.Simulator and cpm.optimisation.FminBound, enables users to conduct full model recovery studies, such as the one shown on Fig 7B. The parameter and model recovery code is included in the tutorials on the documentation website.

### Archiving results

The toolbox organises results into tabular long-format data in pandas.DataFrames, allowing users to save the output of their work in CSV or other supported formats. Furthermore, the toolbox allows users to export key information about the parameterisation of their model, such as lower and upper parameter bounds, and priors placed on the model parameters. For optimisation methods, *cpm* outputs subject-level parameter estimations and related metrics (fit quality or Hessian matrices) organised in tabular format. cpm.generators.Simulator and cpm.generators.Wrapper organise the trial-level model outputs and predictions into a similar tabular data format, ready for further analysis. All function documentations include a description of their outputs and a codebook for the variable when appropriate.

## Availability and future directions

*cpm* is available through the Python Package Index (PyPI) and can be installed by typing pip install cpm in the terminal. The toolbox is completely open source under the GNU Affero General Public License version 3. Open-source software is essential for scientific inquiry, not just in terms of open code to aid in reproducibility, transparency, or the adoption of new methods, but to promote open development and recognition of contributors [97–99]. The development of the toolbox is carried out on GitHub (https://github.com/DevComPsy/cpm), where users can also access pre-release and nightly versions that include the most recent updates and bug fixes. Examples and installation instructions are readily available online on Jupyter Notebooks for immediate use, see the documentation’s website.

### Future directions

One of our primary objectives involves broadening the scope of model categories. The *cpm* toolbox already provides models for learning, risky decision-making, and metacognition, with plans to expand into additional model categories. Numerous model components exist in the current literature, and it is our goal to integrate as many of these elements into the library as possible. Moreover, we aim to incorporate preconfigured models into the library, providing users with ready-to-use applications. In this vein, users can request new features and models through the GitHub portal (see the Contribution section for more information about requesting features.) and ask for support in applying the toolbox to non-canonical experimental tasks. We also envisage that, as usage of the toolbox increases, we will be able to collect user-generated “recipes” into a “cookbook”-style document that will allow new users to find a starting point that is very close to their desired goal.

We intend to introduce a more extensive model comparison and selection module, which will include model selection metrics, such as Bayes Factors [100,101] and Exceedance Probabilities [102], which will build on the hierarchical estimation techniques that we have already implemented. Furthermore, we are also working on implementing model selection metrics that are sensitive to the models functional form, such as Minimum Description Length and other Bayesian model selection criteria [91].

Our plans also include the implementation of novel and more efficient methods for model fitting using Hierarchical and Markov Chain Monte Carlo approaches, such as Variational Bayes Monte Carlo [103,104], and the implementation of sophisticated and large-scale simulation-based approaches to model evaluation, such as parameter space partitioning [44], model evaluation techniques such as landscaping [105], and novel discrete multi-objective optimization approaches, *g*-distance [106]. Beyond feature implementations, our objective is to improve computational time concerning the various model components and the data compilation process.

A major planned improvement is to extend the current infrastructure to integrate *cpm* with BrainExplorer (https://brainexplorer.net/), a smartphone application that enables large-scale cognitive testing in ecologically valid settings, pushing computational modelling beyond traditional laboratory environments. While *cpm* aims to standardises modelling workflows, data acquisition and experiment deployment remain common sources of inconsistency across labs. This integration aims to make datasets analysis-ready by design, dataset versioning more streamlined, and workflows reproducible from collection to inference, and changes traceable. In practice, BrainExplorer would provide the data-side interfaces and collection, while *cpm* provides model specification, estimation, evaluation and reporting utilities.

### Contribution

We highly value contributions of all kinds, including improvements to documentation, reporting and fixing bugs, and implementing or requesting new models and methods.

Users can propose changes or report issues via the Issues page of the GitHub repository, and contribute directly to the codebase through Pull Requests. All contributions are reviewed by the core development team, and extensive test coverage is in place to maintain functionality following any change to the codebase. Detailed guidelines for contributing are provided in the CONTRIBUTING.md file available in the GitHub repository of the toolbox, and a roadmap of the current developmental priorities and planned features is provided on the cpm *documentation website* (see also the Future directions section). All contributors will be listed as developers of the toolbox.

Furthermore, users can ask any questions related to the toolbox, seek help with debugging, or discuss ideas on the GitHub Discussions platform. We actively encourage community engagement, as user feedback helps guide the development of the toolbox and ensures that it meets the needs of researchers.

## Closing remarks

The algorithms and general framework in *cpm*, implemented using a high-level programming language, serve as foundational tools for tailored approaches in theory-driven computational psychiatry. *cpm* to date covers many areas, such as metacognition, probabilistic learning, and risk-based decision-making. By building an overarching, customisable, and accessible software system, we hope to provide a foundation for the wider adoption of and progress in computational methods in Computational Psychiatry specifically, and Psychology / Cognitive Neuroscience more broadly. Reproducibility and transparency are central to this effort, and the toolbox is designed to encourage and support both throughout the modelling workflow. Finally, *cpm* is an open and actively developed project, and we welcome contributions from the community to expand the model catalogue, analysis utilities, and documentation.

## Supporting information

S1 Text The following algorithms are provided in the Supporting Information. (PDF)

S1 Algorithm Delta-rule learner with softmax choice for a *k*-armed bandit. (PDF)

S2 Algorithm Anti-correlated update rule (Eqs. 3–4). (PDF)

S3 Algorithm Kernel update rule (Eq. 5). (PDF)
