# Supplementary material for: cpm: A python library for theory-driven modelling in computational psychiatry
Source: PLoS Comput Biol. 2026 Jul 13;22(7):e1014481. doi: 10.1371/journal.pcbi.1014481 (PMC13379104; doi:10.1371/journal.pcbi.1014481)
Supplement: S1 Text — (PDF) [file pcbi.1014481.s001.pdf]

Supporting Information

Lenard Dome[1,2]*, Frank H. Hezemans [1,2,3,4] Kenza Kadri[1,2], Ben J. Wagner[1,2,3], Andrew Webb[3], Tobias U. Hauser[1,2,5]

1 Department of Psychiatry and Psychotherapy, Faculty of Medicine, University Tübingen, Tübingen, Germany **2** German Center for Mental Health (DZPG), Tübingen, Germany **3** Max Planck Institute for Biological Cybernetics, Tübingen, Germany **4** Institute of Cognitive Science, Osnabrück University, Osnabrück, Germany **5** Max Planck UCL Centre for Computational Psychiatry and Ageing Research, University College London, London, United Kingdom

* lenard.dome@uni-tuebingen.de

Model Specification used in example

Let each stimulus have an associated value, which is the expected reward that can be obtained from selecting that stimulus. Let also $Q(a)$ be the estimated value of action a. In each trial, t, there are two stimuli present, so $Q(a)$ could be $Q(\text{left})$ or $Q(\text{right})$, where the corresponding Q values are derived from the associated value of the stimuli present on the left or right. In each trial t, the Softmax choice rule [1] will convert the estimated value of each action into a probability according to the following policy:

$$P(a_t) = \frac{e^{Q_t(a)\beta}}{\sum_{i=1}^{k} e^{Q_t(i)\beta}} \tag{1}$$

where β is the inverse temperature parameter, also known as choice stochasticity, and $Q_t(a)$ is the estimated value of the action a at time t. k is the number of actions available, which is in our case, $k = 2$. Using this, we can now define the loss function based on negative log-likelihood.

$$-\log L(\theta \mid Y, M) = -\sum_{i=1}^{N} \log \Big[p(y_i \mid \theta) \Big]$$

where θ are model parameters, Y is the data with N number of data points, and M is the model. The $p(y \mid \theta)$ gives the probability of observing the data given a certain set of parameters. In our current applications, it is always the model's probability of choosing the arm that participants selected. Note that the loss function is not part of our model specification, but is implemented in `cpm.optimistaion.minimisation.LogLikelihood.bernoulli`. For tasks with more than two possible actions this can be replaced by `LogLikelihood.categorical` without changing the model implementation.

The model uses the variant of the delta rule [2,3] adapted for multi-armed bandit problems where each option has a single stimulus dimension [4], reducing Rescorla-Wagner's summed error-term to the following equation, similar to 5:

$$\Delta Q_t(A_t) = \alpha \times \Big[R_t - Q_t(A_t) \Big] \tag{2}$$

where α is the learning rate and R_t is the reward received at time t, also called a teaching signal or the target value, and sometimes annotated as λ. A_t is the action chosen for the trial t.

In the model recovery simulations, we included two additional models. One with an anti-correlated learning rule [6] and a kernel choice update rule [7]. The anti-correlated learning rule is defined as

$$\Delta Q_{t,a} = \alpha \times \Big[R_t - Q_{t,a}\Big] \times s_a \tag{3}$$

where t is the current trial and a is the action. R is the reward received. The new addition, s_a, is a vector, where each chosen and unchosen action a at time t is denoted by 1 and -1, respectively. We also imposed the following constraints on the updated Q-values:

$$Q_{t+1}(A_t) = max(0, Q_{t+1}(A_t)) \tag{4}$$

In most standard applications, Q-values can be negative. For our demonstrations we constrained them to a minimum 0 – corresponding to the absence of reward and the lowest possible outcome R. In some trial orders with long runs of the same stimuli, or if participants have strong preferences for other objects over a large number of trials, Q-values may diminish to excessively large negative figures, which is something we wanted to avoid in order to have a more stable and constrained model behaviour for these demonstrations. The kernel update rule is defined following [7]:

$$\Delta Q_t(A_t) = \alpha\Big[(R_t - \alpha) - Q_t(A_t)\Big] \tag{5}$$

To make the model specification above more concrete, S1 Algorithm summarises the full trial-by-trial procedure as pseudocode. It combines the three components introduced in the preceding section: the softmax choice policy (Eq. 1), the delta-rule value update (Eq. 2), and the negative log-likelihood loss used for parameter estimation. On each trial, the learner converts the current action values into choice probabilities, accumulates the log-likelihood of the participant's observed choice, and then updates the value of the chosen action using the reward prediction error. Only the chosen action's value is modified; unchosen values are carried over unchanged. The procedure takes the learning rate α and inverse temperature β as its only free parameters, and returns the negative log-likelihood $\mathcal{L}(\theta \mid Y, M)$ that is minimised during model fitting. S2 Algorithm and S3 Algorithm for the other two model variant.

References

1. Bridle JS. Probabilistic interpretation of feedforward classification network outputs, with relationships to statistical pattern recognition. In: Neurocomputing: Algorithms, architectures and applications. Springer; 1990. p. 227–236.

2. Rescorla RA, Wagner AR. A theory of Pavlovian conditioning: Variations in the effectiveness of reinforcement and nonreinforcement. In: Black AH, Prokasy WF, editors. Classical Conditioning II: Current Research and Theory. Appleton-Century-Crofts; 1972. p. 64–99.

3. Rumelhart DE, Hinton GE, Williams RJ. Learning representations by back-propagating errors. nature. 1986;323(6088):533–536.

4. Barto AG, Sutton RS. Reinforcement learning: An introduction. 2nd ed. The MIT Press; 2018.

5. Bush RR, Mosteller F. A mathematical model for simple learning. Psychological review. 1951;58(5):313.

6. Hauser TU, Iannaccone R, Stämpfli P, Drechsler R, Brandeis D, Walitza S, et al. The feedback-related negativity (FRN) revisited: new insights into the localization, meaning and network organization. Neuroimage. 2014;84:159–168.

7. Wilson RC, Collins AG. Ten simple rules for the computational modeling of behavioral data. Elife. 2019;8:e49547.
