# Supplementary material for: cpm: A python library for theory-driven modelling in computational psychiatry
Source: PLoS Comput Biol. 2026 Jul 13;22(7):e1014481. doi: 10.1371/journal.pcbi.1014481 (PMC13379104; doi:10.1371/journal.pcbi.1014481)
Supplement: S1 Algorithm — (PDF) [file pcbi.1014481.s002.pdf]

S1 Algorithm. Delta-rule learner with softmax choice for a k-armed bandit

Require: Trials $\{(s_t, y_t, R_t)\}_{t=1}^{N}$, learning rate $\alpha \in [0,1]$, inverse temperature $\beta \geq 0$

Ensure: Negative log-likelihood $\mathcal{L}(\theta \mid Y, M)$ with $\theta = \{\alpha, \beta\}$

Initialise $Q_1(a) \leftarrow 0$ for all actions $a \in \{1, \ldots, k\}$
$\mathcal{L} \leftarrow 0$
for $t = 1$ to N **do**
  $\mathcal{A}_t \leftarrow$ actions available on trial t ▷ e.g. $\{\text{left}, \text{right}\}$
  for each $a \in \mathcal{A}_t$ **do** ▷ Softmax policy, Eq. (1)
    $u_t(a) \leftarrow \exp\big(\beta\, Q_t(a)\big)$
  end for
  $Z_t \leftarrow \sum_{a \in \mathcal{A}_t} u_t(a)$ ▷ Normalising constant
  for each $a \in \mathcal{A}_t$ **do**
    $P_t(a) \leftarrow u_t(a) \,/\, Z_t$
  end for
  $A_t \leftarrow y_t$ ▷ Action chosen by the participant
  $\mathcal{L} \leftarrow \mathcal{L} - \log P_t(A_t)$ ▷ Bernoulli/categorical NLL
  $\delta_t \leftarrow R_t - Q_t(A_t)$ ▷ Prediction error
  $Q_{t+1}(A_t) \leftarrow Q_t(A_t) + \alpha\, \delta_t$ ▷ Delta-rule update, Eq. (2)
  for each $a \neq A_t$ **do**
    $Q_{t+1}(a) \leftarrow Q_t(a)$ ▷ Unchosen values unchanged
  end for
end for
return $\mathcal{L}$
