# Supplementary material for: cpm: A python library for theory-driven modelling in computational psychiatry
Source: PLoS Comput Biol. 2026 Jul 13;22(7):e1014481. doi: 10.1371/journal.pcbi.1014481 (PMC13379104; doi:10.1371/journal.pcbi.1014481)
Supplement: S2 Algorithm — (PDF) [file pcbi.1014481.s003.pdf]

S2 Algorithm. Anti-correlated update rule (Eq. 3–4)

for each $a \in \mathcal{A}_t$ **do**

$$s_a \leftarrow \begin{cases} +1 & \text{if } a = A_t \\ -1 & \text{otherwise} \end{cases}$$

$$Q_{t+1}(a) \leftarrow Q_t(a) + \alpha \left(R_t - Q_t(a)\right) s_a$$

$$Q_{t+1}(a) \leftarrow \max\left(0,\, Q_{t+1}(a)\right)$$ ▷ Non-negativity constraint

end for
