# Supplementary material for: cpm: A python library for theory-driven modelling in computational psychiatry
Source: PLoS Comput Biol. 2026 Jul 13;22(7):e1014481. doi: 10.1371/journal.pcbi.1014481 (PMC13379104; doi:10.1371/journal.pcbi.1014481)
Supplement: S3 Algorithm — (PDF) [file pcbi.1014481.s004.pdf]

S3 Algorithm. Kernel update rule (Eq. 5)

1: $Q_{t+1}(A_t) \leftarrow Q_t(A_t) + \alpha \left[(R_t - \alpha) - Q_t(A_t)\right]$
