# Supplementary material for: cpm: A python library for theory-driven modelling in computational psychiatry
Source: PLoS Comput Biol. 2026 Jul 13;22(7):e1014481. doi: 10.1371/journal.pcbi.1014481 (PMC13379104; doi:10.1371/journal.pcbi.1014481)
Supplement: Attachment — Submitted filename: ploscompbio-cpm-paper-review_final.pdf [file pcbi.1014481.s005.pdf]

Summary

The authors introduce the Computational Psychiatry Modelling (cpm) toolbox, a Python library for theory-driven modelling in computational psychiatry and cognitive (neuro-)science. The communicated primary goal of the toolbox is to provide a single, accessible, and overarching framework that integrates a wide range of established computational approaches under one roof. cpm goes beyond purely model building or an exclusive focus on parameter estimation and instead houses a more comprehensive workflow. The authors highlight that this standardisation is crucial for overcoming current cultural blockers in the discipline, which has a tendency toward redundancy via e.g. unnecessarily disparate, lab specific modelling practices and code-bases that are not interoperable. This creates substantial challenges for non-expert researchers from backgrounds like psychiatry and psychology, who find it difficult to navigate the space and contribute to research in computational psychiatry.

cpm aims to cover various tasks, some of which are mentioned explicitly such as risky decision-making, reward/punishment learning, and perceptual metacognition, with associate reinforcement learning methods as well as signal detection theory. Core functionalities include model building via reusable components, automated subject-level parameter estimation, methods for generic model evaluation as well as standardized reporting of results. By relying on the scientific computing stack in python, the toolbox supports a reasonably broad array of downstream optimization methods. The authors argue that cpm will help consolidate computational approaches in the field of computational psychiatry.

Major Comments

While I am in full agreement on some of the fundamental motivators for tools, such as cpm, the exposition currently has some weak points, which I will try to list below:

First, from the exposition in the paper it remains unclear how a generative model is specified in its entirety. Is what the user writes the equivalent of a likelihood or a simulator? Both pieces are important, and can look like very distinct code (in fact, in most non-trivial cases, the simulator code and the likelihood code wouldn’t resemble each other to the point where inferring one from the other seems impossible). Usually it is the likelihood that underlies the objective function used for optimization and the simulator that is used for e.g. prior and posterior predictives.

The distinction is made only quite weakly in the text. “components” of models (e.g. in the cpm.models module) seem to refer specifically to the likelihood part. However arguably a “model” can reasonably be defined first and foremost as a simulator.

From the flow chart in Fig 1., simulators seem to be associated only with the last step in the workflow. This has implications for the class of models that are taken into account a priori (see my points below concerning discussion toolboxes that focus on simulation based inference, such as BayesFlow and the sbi toolbox in Python).

Second, the construction of a model in Listing (4) looks too much like ad-hoc code, from which it is hard to deduce specific principles about how to approach a user contribution. You might be better served by adding a pseudo-code example that illustrates conceptual building blocks with less specific details.

Third, (this is again emphasized below in response to a specific statement in the paper), the authors include too sparse a discussion of the existing software eco-system. Two axes should be explored and differences and similarities should be discussed in more detail. Axis one being similar tools with a priori somewhat similar goals, such as HSSM, EMC2. Axis two is a nod to adjacent toolboxes such as BayesFlow and sbi, which vastly expand the feasible mode space and provide a take on alternative overall modeling workflows. To aid readers in their approach to computational modeling in computational psychiatry and beyond, it is important to provide a sufficient level of contextualization along both axes.

Fourth, too vague of a discussion of more technical details that leave the reader guessing. I will give two examples:

1. The term “compiled” is used without a clear explanation of what is meant in this context. This ties in with the point below concerning differentiability.
2. The term “large-scale simulations” is used without explaining the core mechanic that allows the toolbox to assemble trial-wise code such that simulation is efficient downstream.

Fifth, nowhere does the paper include a discussion of differentiability of likelihood functions. This is an important, de facto crucial concept for model construction and the scope of inference algorithms that can be applied downstream (sampling and/or optimization). E.g. can models be written and “compiled” (due proper clarification of the term as per above) as JAX function etc.. While it is not strictly necessary for this toolbox to be useful to the community, a discussion of this aspect through the lens of the ambition, extensibility of the toolbox etc. however is. I consider it crucial in the contemporary landscape of popular techniques in computational modeling as well as machine learning.

Sixth, relating to the standardization of workflows and reproducibility of computational experiments in computational psychiatry, as motivated in the introduction, the article could benefit from highlighting the specific features of the package that allow loading and saving of the models (eg. parameterization instantiations), meta-data (eg. task paradigm constants), experimental settings (eg. optimization settings), and results (beyond saving the traces or diagnostic figures).

Seventh, it is unclear whether the toolbox would support (or aims to support) incorporation of other task paradigms employed in computational psychiatry moving beyond the canonical learning or decision-making tasks - for example, trust games, cognitive control tasks, etc.

Reaction to specific quotes:

1. The authors comment (Line 48-49), that “existing toolboxes that cover computational methods used in the field tend to be special-purpose, with a strong focus on one type of model and a lack of flexibility in model building”.

This cites HDDM, HBayesDM and Piray et. al. (2019). Two comments:

First, the citations should be expanded to include other toolboxes such as EMC2, HSSM and RLSSM and PyDDM (which is mentioned somewhat later in the paper in a different context).

Second, specifically the HSSM eco-system, which this reviewer is involved in developing, is explicitly designed to foster community contributions centered around new models into a shared repository (while other aspects of emphasis may differ). The stated limitation of toolboxes in the field should be somewhat attentenuated.

Concerning the first point, please expand the citations to provide a broader picture of the existing eco-system. Concerning point two (which comes up in other comments above) please provide some level of description of the differences between what you are doing and what a tool like HSSM provides.

2. In lines 124-126 the authors claim that “a significant distinction between cpm and other tools employed in Computational Psychiatry is its capacity to assist users in both constructing models and estimating parameters… [...]”

In a similar vein to the above comment concerning the sparse representation of the existing software eco-system, the authors should attenuate this claim and possibly contrast more closely with tools like HSSM (and it’s wider eco-system) which do take these thoughts into account (albeit manifesting quite differently).

Minor Comments

Line by line:

Line 12:
“Using such an approach…” → “This approach”

Line 20-22:
Run on sentence, could be formulated to be a bit more crisp

Line 31:
“For them” → too informal

“, and” → start new sentence

Line 46:
Quotation marks need to be corrected

Line 54-:
“We believe that such an open-source open-access toolbox” → in light of major comments concerning the slight underrepresentation of the toolbox eco-system, I would consider it more appropriate to us phrasing as: “... such open-source open-access toolboxes” to highlight the general point without insinuating uniqueness as strongly

Line 57-74:
The difference between the description of modularity and flexibility is somewhat weak. Flexibility seems to essentially follow from modularity here. Maybe flexibility is rather described as interoperability?

Line 65:
“The toolbox” → surrounding sentences use “the toolbox” too many times, try to reduce repetition

Line 79:
“have distinct purposes” → “have a distinct purpose”

Line 108:
“with concrete example” → “with concrete examples” ?

Line 111:
“To any scientists/researchers” → … “in the domain of [...]”

Line 114:
“[...], where” → make it new sentence

Line 132-133:
“[...] that are implemented as stateful list processors” → Somewhat unmotivated terminology. Since “stateful list processors” comes up a few times, it might be worth actually explaining what exactly this concept is about.

Line 144:
“In such cases, the toolbox compiles” → I was unable to dissect what exactly is meant by the term “compiled” in this paper. Is this the technical use of the term, as in “compiled code”?

Line 186:
“[...] their own models, either from scratch or [...]” → break up sentence

Line 260:
“encapsulates the model with Wrapper” → “encapsulated the model with the wrapper class”? Wrapper is also a very generic term, possibly consider renaming the functionality.

Line 266:
“Wrapper objects can also serve as breakpoints, [...]” → endpoints? Somewhat unusual phrase and specifically in the context of software, mostly used when talking about debugging.

Line 287-289:
“[...], and are repurposed here to avoid codebase fragmentation that would lead to unnecessary divergence - more informally, we do not with to ‘reinvent the wheel’” → Can be formulated positive as e.g. “we use battle-tested code from established libraries that are part of the scientific computing stack in python”

Line 311:
“Cpm.optimisaion.compare” → typo

Line 324:
“into a hierarchical one” → “into a hierarchical counterpart”

Line 325:
“The users have already defined [...]” → “a user was already prompted to …”

Line 326:
Again, the use of the term “compiled” is a bit unclear.

Line 332:
“Data-driven techniques that directly estimate hyper-parameters for hierarchical models from empirical data, namely Hierarchical Bayesian Inference based on Variational Bayes [...]” → Slightly unclear use of terminology. In principle Variational Inference is not a “counterpart” to “Empirical Bayesian Estimation”. Variational Inference is just an optimization based approach to estimate probability distributions (posteriors in the standard sense of Bayesian Inference). It doesn’t imply anything specific about directly estimating “hyper-parameters” [...] from empirical data.

Line 346:
“The Simulator class streamlines large-scale simulations, [...]” → “large-scale” is used too generically.

Line 353:
“[...], which users can then use” → “[...], which can then be used” e.g.

Line 359:

“All of this was done without leaving the toolbox or relying on large amounts of error-prone code” → too vague

Line 385:
“We want to make it clear that if you decide to contribute, our goal will be to help you make the best contribution possible” → drop sentence

Line 387-389:
“We have had very positive experiences with people who contributed substantially to previous packages, often resulting in large contributions as part of collaborative research projects” → Should be reformulated as a contribution guideline, this sentence as is is more appropriate for a grant application than a paper.

Line 426-428:
“The toolbox currently does not aspire to cover Drift Diffusion Models, as these are already comprehensively supported by the Python library, PyDDM [...]” → Should cite HDDM, HSSM, EMC2 (and possibly more) here. Moreover, it is unclear what “not supporting” means in the context of the story of the paper. Not attempting to include Drift Diffusion Models as a core model with examples in the codebase? Or is there a fundamental notion as to how it would be difficult to support these models?

General:

Sometimes empty lines are used after e.g. imports in the codeblocks and sometimes not. Make formatting homogeneous on that front.
