# Supplementary material for: cpm: A python library for theory-driven modelling in computational psychiatry
Source: PLoS Comput Biol. 2026 Jul 13;22(7):e1014481. doi: 10.1371/journal.pcbi.1014481 (PMC13379104; doi:10.1371/journal.pcbi.1014481)
Supplement: Attachment — Submitted filename: Rebuttal Letter.pdf [file pcbi.1014481.s007.pdf]

Editor's Comments :

COMMENT#1: Thank you very much for submitting your manuscript to PLOS Computational Biology. All reviewers agreed that your study makes a valuable and original contribution to the field. In particular, they commended the scientific motivation and potential impact.

At the same time, the reviewers identified several important issues that will need to be addressed before the manuscript can be considered for publication. The main concerns relate to […] (1) the clarity and completeness of the modeling description and its relation to existing approaches […]

RESPONSE: We thank the editor for their guidance and feedback on our paper and the helpful suggestions. W**e implemented these changes and directly respond to** all reviewer raised comments a below. These suggestions have helped us to make the paper more coherent, and we believe that the revised version now offers a much more rounded presentation of the toolbox.

Concretely, we have revised the entire manuscript to clearly separate the technical modelling details from the user-facing walkthrough. We further revised the modelling description to be more explicit and self-contained. The technical specifications, design and implementation choices are presented in the **Design (page 4, Line 110)** and **Implementation (page 6, Line 193)** sections, while the walkthrough is presented later as an accessible, end-to-end example. We also expanded the direct comparisons of cpm to other tools in terms of scope, extensibility, and supported workflows, resulting in the new **Related Work (page 15, Line 478)** section, that now cites all works recommended by the reviewers.

COMMENT#2: […] (2) difficulties encountered in running the provided code.

RESPONSE: We apologise for the limited accessibility of the code in the previous submission. In response to this **point**, we have substantially revised the codebase and now provide a refined version available at the following **URL**. We have tested this version across various operating systems and are confident it will run reliably across user environments.

Reviewer #1:

COMMENT#1: This manuscript presents cpm, a Python library for theory-driven computational modeling in psychiatry and cognitive neuroscience. The toolbox integrates a wide range of modeling approaches (reinforcement learning, associative learning, signal-detection, and hierarchical Bayesian estimation) in a unified framework intended for both novices and experts. The paper offers a walkthrough of cpm's architecture, coding principles, model construction, optimization, hierarchical estimation, and simulation capabilities. I have co-reviewed this article with a graduate student in my lab. On the whole, the toolbox addresses the important need in computational psychiatry to standardize model-fitting procedures and cut down on error-prone custom code across projects.

RESPONSE: We thank the reviewers for their thorough and generous assessment of our effort and for taking the time to co-review the manuscript. We are pleased that the reviewers recognise the need for standardised model-fitting procedures in computational psychiatry and share our view that reducing error-prone custom code across projects is an important goal. We have carefully addressed all concerns raised and provided a detailed point-by-point response below. We hope the revised manuscript and toolbox meet the reviewers' expectations.

COMMENT#2: Although we see considerable strength in the direction of the toolbox, the manuscript could be made stronger in terms of its didactic value and clarity, as well as its implementation. The current manuscript reads more like a developer's overview than a didactic guide or validation paper. Clarifying the intended user, expanding empirical benchmarking, and restructuring the examples could greatly improve accessibility and impact.

We saw two problems in the construction of the paper: 1) the examples/listings do not flow in a linear way that would serve as a 'quick start' tutorial for a new user and give a sense of increasing complexity (from off-the-shelf to fully custom), and 2) the scope of models encompassed by cpm is not clear, making it hard for a reader to know if they should consider the package further.

RESPONSE: We thank the reviewers for their very helpful analysis of the didactic approach and clarity of our original manuscript. We have now entirely revised the manuscript to improve the didactic value and clarity. We have comprehensively revised the manuscript to clarify our intended audience and restructure the flow to accommodate both beginners and more advanced coders. We provide a more detailed response to each point below.

COMMENT#3: In terms of the first concern, the example seeks to go from fully built to more customized, and this makes sense. But most of the transitions are stated in technical terms, like "We specify two free parameters: alpha for the learning rate, and a temperature parameter governing the choice stochasticity." Rather, such transitions should be explained more like, "Although we used default parameter values of X and Y for learning rate and temperature in the cpm.applications example above, what if we wish to specify different priors on the learning rate and temperature?" Similarly, when going to greater levels of complexity (e.g., specifying the model in Listing 4), the reader needs more of a checklist, like, "As the user moves from fully built models such as cpm.applications.reinforcement_learning.RLRW to more customized ones, they must specify the core ingredients of a model within the framework. In particular, as shown in Listing 4, the model definition must include..." Rather than stay the didactic level, the paper is too eager to communicate its breadth and novelty, like, "Thus, we put extra effort into these features of the toolbox, making the parameterisation of models within cpm robust, reliable, and equipped with numerous functionalities."

RESPONSE: We thank the reviewers for these concrete and helpful suggestions. We have now revised the walkthrough to make sure it serves a **user-friendly** tutorial beginning on page 16. Specifically, we now use reader-motivated, non-technical transitions to guide users from fully-built examples to customisable models. We also motivate the custom parameterisation by giving examples of why a user might need to adjust them (changing parameter bounds or priors); and added a short ingredients checklist on what users must define when moving from fully built to custom models.

In addition, we softened the phrasing in the walkthrough in favor of a user-oriented didactic language. For example, we included introductory text for each step in the walkthrough to motivate the next steps we take in the modelling workflow. One concrete example is the **Making Models Executable** section on page 20 (beginning on line **698**): *"Once the parameters and user-written model function are specified, the immediate next steps are usually practical: does the model behave sensibly, do the trial-by-trial calculations match what we think the model should be doing, and can we apply it to the data to inspect the learning curves?"*

COMMENT#4: To summarize, I strongly recommend separating a clear walkthrough of the RLRW/Q-learning model from efforts to describe the scope of the package and some of the interesting, but not

directly relevant, architectural details. After the reader has a worked example in hand, including why they might wish to swap out the softmax choice rule for another, you can then transition to talking about things in more general terms, clarifying the architectural principles and major modules of cpm. At present, there are far too many asides in the example that distract, such as, "Examples include Q-values in reinforcement learning models [39], or transition matrices in model-based and model-free learning. These methods are often used internally by the library; for example, calculating the probability density functions (PDF) for priors is used to automatically turn any model into a hierarchical one without expecting the users to hand-code the transition."

RESPONSE: We thank the reviewers for their helpful suggestion. We have implemented such a separation in the revised version of our manuscript as follows:

1. We have now separated the didactic walkthrough from the description of breadth by delegating the technical details to the **Design (page 4, Line 110)** and **Implementation (page 6, Line 193)**.

2. The novel contribution of the toolbox is now discussed in comparison to other tools in the newly added **Related Work (page 15, Line 478)**.

We hope this change of organisation is in line with the reviewers' ideas and has helped the readability.

COMMENT#5: Second, although we appreciate that it may be difficult to provide an exhaustive list of the packages scope, I believe you should try to try to include something on this matter. For example, consider including a table that lists the models available -- or even the major families. For example, there is already a clear "learning model" category inherent to the RLRW Wrapper. It would help the reader to spell out these categories more explicitly and give rationale and a plan for including categories in the future as the package expands.

RESPONSE: Following this suggestion, we have now specified the existing model categories and planned expansions in the **Scope (page 3, Line 67)** section. Briefly, the toolbox currently has three categories of models available: reinforcement learning, decision making, and metacognition. We note that although learning models incorporate decision processes, we organise models around the phenomena they target rather than their components. In the near future, we plan to introduce a new category of effort-based learning and decision making. We also explicitly mention that the choice of models to implement is driven by the users, who can make a feature request on GitHub.

COMMENT#6: Likewise, what are the 'Legos' that users can reach for when building models and what will they have to code by hand? Without some exposition of the scope (it's currently rather promissory in the text), interest in the package may be limited.

RESPONSE: We thank the reviewers for this helpful point. We have revised our explanation: the newly introduced **Model Execution Framework (page 10, Line 290)** section now begins by explaining what these building blocks are, while our walkthrough explains how to use them to build a model and what to do in cases where we did not implement the equations of interest.

COMMENT#7: At the conceptual level, our biggest concern is that the novelty of cpm relative to prior efforts is somewhat unclear. I believe the novelty lies in the modular approach. I also found the prior = True automation intriguing and novel. Although you emphasize these, there are no direct comparisons to prior efforts (e.g. hBayesDM), including areas where those efforts may be preferable for some applications. For example, hBayesDM gives full MCMC for many common off-the-shelf models where applied users may have no particular motivation to innovate. Consider including a comparison table or

summary in terms of package scope, flexibility/modularity, and estimation and model comparison functionality. More broadly, provide a clearer justification of how cpm can improve reproducibility and model comparability across labs compared to prior packages.

RESPONSE: Thank you for this suggestion. We have now included a **Related Work (page 15, Line 478)** ection that highlights differences between cpm and other tools in terms of novelty, scope and modelling approaches. Throughout the manuscript, we press how cpm helps achieve reproducible workflows. We also specifically mention how it helps with model comparison by enforcing specific data processing schemes via the Wrapper class in the **Core Components (page 8, Line 218).** In addition, we introduced a **Reproducibility Utilities (page 6, Line 178)** section in **Design (page 4, Line 110)** to help pin-point exactly what design choices support reproducible workflows:

"Reproducible modelling starts with a clear and machine-readable representation of trial-level information. In combination with the enforcement of transparent modelling practices, such as defining priors, exporting important details about the optimisation routines, and reporting modelling decisions, cpm both encourages and contributes to reproducible end-to-end workflows.

Archival utilities are also built in to promote reproducible workflows. For example, export functions accompany both parameter management systems and optimisation procedures. They are designed to record additional information about parameters and optimisation routines, convert it into tabular data format that can be saved as a plain text file. Parameter exports support outputting bounds and prior specifications, while optimisation routines likewise record extensive metadata, including warnings, Hessian matrices, goodness-of-fit metrics, iteration counts, and termination criteria. More generally, cpm stores metadata throughout the workflow wherever possible, so analyses can be inspected, shared, and rerun with minimal ambiguity."

COMMENT#8: Second, although the toolbox has "computational psychiatry" in the title, it was unclear to us why this is so. Some useful justification is provided by, "that researchers – especially in Computational Psychiatry - estimate model parameters for individuals as opposed to group-level data." Yet more broadly, the toolbox contain many theory-driven cognitive computational models. To make it truly CP-specific, we would have anticipated new tools or methods for individual difference analysis. For example, some studies have considered whether to fit parameters by clinical group (e.g. Mkrtchian et al., 2017), while others have considered how best to compare parameter estimates across groups or how to link computational parameter estimates with more traditional self-report measures of symptoms or traits. cpm seems explicitly focused on within-person processes, not between-person differences in those processes. Greater clarity on the choice of naming and the omission of individual difference analysis tools would be welcome. Are there plans to develop some individual difference analysis tools that would make it relevant to CP per se?

RESPONSE: This is indeed a fair point. The toolbox was conceived in a Computational Psychiatry lab and assumes subject-level fitting as a foundation for individual difference analysis, which avoids averaging artefacts. However, we agree that dedicated tools for individual difference analysis — such as linking parameter estimates to symptom measures or comparing estimates across clinical groups — are a natural and important extension. We are actively developing in this direction, drawing on recent methodological advances in the field (e.g. Schurr et al., 2024; Moutoussis, Hopkins, & Dolan, 2018). We now clarify this in the manuscript in **Optimisation and Inference (page 12, line 370**). Furthermore, following the reviewer's suggestion, we have added the Mkrtchian et al., 2017 citation in relation to estimating group-level hyperparameters within clinical and control groups separately **(Hierarchical models cpm.hierarchical section, page 24, line 731**.

COMMENT#9: At the technical/implementation level, I had a few suggestions as well. First, it would be helpful for to clarify whether cpm implements unit tests to avoid regression bugs and related issues.

RESPONSE: Yes, we do. Namely, we implemented a suite of regression tests designed to catch bugs and silent breakage introduced by changes to the codebase. We have added a dedicated **Testing and Stability (page 14, line 463)** section to the manuscript detailing our test coverage. The relevant excerpt is provided below:

"To ensure correctness and stability, cpm is accompanied by an extensive suite of automated unit tests implemented with pytest. These tests cover deterministic numerical components (e.g. learning rules), objective functions used during fitting, and ready-to-use model implementations. They also include checks for edge cases (e.g NaN/Inf inputs, shape mismatches, etc.) to ensure robust performance under a wide range of conditions…"

COMMENT#10: Relatedly, does cpm run benchmark tests of results against established implementations of the same model (e.g., in hBayesDM)? These can provide reassurances for the user that each of the core models in the package are trustworthy even as the cpm enables more customization (I appreciate that it would be impossible to create tests for all combinations).

RESPONSE: We agree that benchmark comparisons are valuable features. Although we have not yet released a formal benchmark suite, we have conducted internal cross-validation during development. Specifically, hierarchical estimation routines were cross-referenced against the mfit toolbox (Gershman, 2016; https://github.com/sjgershm/mfit) and the emfit toolbox (Huys et al., 2017; https://github.com/mpc-ucl/emfit) during development, meta-d' model outputs were compared against the HMeta-d toolbox (Fleming, 2017; https://github.com/metacoglab/HMeta-d), and recovery properties of the hierarchical methods were tested on simulated data. These internal checks informed the current codebase but are not yet documented as reproducible public benchmarks. We plan to formalise a small suite replicating 3–5 published results with locked versions and expected outputs in a future release. Cross-implementation benchmarks further require alignment of parameterisations, priors, likelihood conventions, and task definitions, which represents substantial engineering and curation effort beyond the scope of the current paper. In the current release, correctness is additionally supported via unit tests, continuous integration, and simulation-based parameter recovery on canonical cases.

COMMENT#11: Second, the details of the hierarchical prior=True implementation are somewhat unclear. The paper states "During the parameterisation of the model, the users have already defined priors for their parameters, so setting prior=True in the optimisation functions will allow the model to be compiled into a hierarchical model." Although we indeed see information on priors in Listing 3, very little was said about the selection of priors (a big topic, so I'm not suggesting you need to be comprehensive) and it was unclear how these enter into the hierarchical modeling. For example, the Piray HBI approach is rather different from traditional hierarchical modeling in that parameter estimates are informed primarily by those likely to belong to/be described by a given model in the set. Please provide additional detail about how prior=True affects model implementation and how this differs between estimation algorithms.

RESPONSE: We agree that the original phrasing was unclear and have revised accordingly. We clarified in the **Optimisation and Inference** (**Hierarchical Estimation section** beginning on page 12, Line 390) that the setting 'prior=True' augments the fitting by adding the log-prior term to the fitting objective – the fitting is performed on the log posterior (log-prior plus log-likelihood). In standard optimisation, this

setting will regularise the parameters under the specified distribution. In hierarchical routines, these priors are defined as group-level distributions, and their corresponding hyperparameters are estimated from data, which then similarly regulariises individual estimates. We also explicitly note that we currently do not implement the multi-model HBI variant with responsibility-weighted updates across candidate models; this extension is under development and will be released once validated. Finally, because we agree that choice of prior is an important topic, we added some brief guidance and pointers to relevant references for selecting informative priors for cognitive models.

Concerning the selection of priors, we have included additional guidance on selecting priors (Line 434 – 440) and added a reference to Lee and Vanpaemel (2018), which we found to be a key resource on how to provide informative priors for cognitive models.

COMMENT#12: Finally, one of the biggest pain points for users adopting a new package is understanding its data structure expectations -- for example, what should this matrix contain, does that have to be a column vector, do I have to specify a lower bound, will the code blow up if I provide doubles instead of integers? Very little is said about this, yet the code listings reveal what could be strict expectations (e.g., .astype(int) in the stimulus definition of Listing 4).

RESPONSE: Thank you for this suggestion. We agree this is an essential part of the workflow and deserves more explanation. We now discuss the exact data structure expectations in the new **Data Format and Processing (page 11, Line 337)** section and elaborate on how the Wrapper operates on trial-level information in the **Model Execution Framework (page 10, Line 290)**:

"*Custom models in cpm are created by defining a trial-level model function and wrapping it with cpm.generators.Wrapper. The model function must operate with a signature model(parameters, trial, ...), where parameters is a structured cpm.generators.Parameters object; and trial is typically a pandas.Series (or a dictionary) containing the task inputs for that trial. The model function returns a dictionary containing at minimum a dependent entry: the model-generated prediction that will be compared against observed in the loss function (e.g., the probability of the observed choice). The function may also return updated state variables; any returned keys that match parameter names are automatically carried forward to the next trial. This design supports both fitting (using an observed column) and simulation (sampling choices from the model's policy).*"

COMMENT#13: Likewise, several structure-related Pythonisms/conventions are embedded in the listings but not described. For example, the .flatten() method in Listing 4, or the .copy of Qvalues (Listing 4), or .groupby('ppt') in Listing 6. Please provide more guidance on the key inputs to the package and whether the package is tolerant of variation (e.g., whether it would convert to int internally for a multinomial outcome etc.)

RESPONSE: We agree that this is an important point. We have now added further explanations as comments to the code to clarify the Python-ism conventions.

Additional comments:

COMMENT#14: For applied users without a deep background in mathematics and programming (which cpm intends to serve), there seems like a large gap between using a pre-defined Wrapper and building a model from scratch. While the package provides basic functions for the latter, the code in Listing 4 is still somewhat tedious and may be inaccessible to many users. Is there a way to find a middle ground? For example: the lines in Listing 4 that call pre-defined functions in the package are clearly the important "modular" elements that are meant to be the heart of the package (e.g., `DeltaRule` versus

`SeparableRule`, etc.). Could the developers add an argument to the RLRW Wrapper such as "learning_rule" that then the user could specify which of those sub-functions is being called? This would give the user the flexibility you seek but keep the non-computational user from having to copy and paste the base code and edit it.

RESPONSE: We agree there is a large gap between fully prebuilt applications and defining a model from scratch. While we have considered a single highly configurable Wrapper (e.g. learning_rule=), implementing arbitrary model variants within one class is not feasible due to combinatorial growth in options and the resulting complexity in the parameter initialisations and the tracking of latent variables, documentation, validation and test units. In particular, swapping a learning rule often results in a change of priors, initialisation, and latent-state structure, so a simple flag would introduce substantial hidden changes and a large testing surface.

Instead, we provide a middle ground through curated and validated templates as fully-built applications such as RLRW and improved customisability (custom model functions, model skeletons, coded equations), so users can make as minimal modification to the implementations as they wish. We also envisage that, as usage of the toolbox increases, we will be able to collect user generated "recipes" into a "cookbook"-style document that will allow new users to find a starting point that is very close to their desired goal. Furthermore, cpm already ships with multiple ready-to-use model implementations and an extensive set of worked examples, which provide practical starting points for users at all levels of expertise. Nonetheless, we keep your comments in mind and continue to explore ways to reduce this gap. We have now added this to the **Future Directions (page 29, line 821)**.

COMMENT#15: pg. 7 The RLRW abbreviation is never defined.

RESPONSE: The RLRW abbreviation has been defined upon first use in the revised manuscript.

COMMENT#16: Listing 4, Line 28-30 Why isn't this done by the function or at least given as an option through an argument to the function? (Pertains to broader questions about data structure and tolerance of violations)

RESPONSE: We have already implemented this check within the function, where we now check function arguments and latent variables as well. We have started implementing these changes throughout the codebase, so we adapted the code in the paper to match the latest version of the toolbox.

COMMENT#17: Although we appreciate the importance of not being redundant with other packages, not including DDM in some form seems like a weakness. Is there a way to wrap the PyDDM package into this one? Since you emphasize standardization/a unified approach I see this as being an opportunity to bridge the divide between DDM modelers and others in terms of software development, etc. Relatedly, is there support for RL-DDM approaches?

RESPONSE: We agree that DDMs are a central part of the modeling ecosystem and excluding them might look like a gap. We also agree that standardization is an opportunity here, and the most sensible way forward is to work on interoperability between these packages. We are currently exploring what such an implementation might look like in future releases. We currently don't ship dedicated RL-DDM models as part of the core package. We believe that our new **Related Work** (page 478, line 511) and **Scope** section (page 3, line 66) clarifies this scope. Briefly, we decided to not cover DDM approaches because these are already comprehensively supported by a number of complementary packages. This reflects a choice in our scope and not a fundamental incompatibility.

COMMENT#18: Model comparison is largely set to the side as a separate topic, but could potentially be incorporated into the walkthrough. On p. 10, this seems like a place to segue into the description of model comparison, "In this case, the generic cpm.models.learning.Softmax class includes this version as an associated method, so we just exchange choice_rule.compute() on lines 24 to choice_rule.irreducible_noise(). This allows users to explore how thematically related variants of the same computation impact model fits and behaviours. In terms of general software architecture, this approach lets cpm organise these modifications as part of the same class for ease-of-use and theoretical alignment"

RESPONSE: We agree that the walkthrough's discussion of swapping is a natural point to motivate model comparison, and we now added a mention how these variants promote model comparison in the **Specifying processes with cpm.models section (page 19, line 649).** The toolbox provides standard model comparison metrics, including BIC and AIC, which users can apply to evaluate competing models fitted within the same workflow. We have also adjusted the **Model Comparison section (page 22, line 722)** to describe the available functionality and outline planned extensions, including tighter integration of comparison metrics within the optimisation workflows.

Reviewer #2:

Major Comments

COMMENT#1: While I am in full agreement on some of the fundamental motivators for tools, such as cpm, the exposition currently has some weak points, which I will try to list below:

RESPONSE: We thank the reviewer for their in-depth evaluation of our manuscript and for the many helpful concrete suggestions on how to further improve it. **Below, w**e address each of the raised points in detail how we revised the manuscript accordingly. We hope our revised manuscript now fulfils the expectations of this reviewer.

COMMENT#2: First, from the exposition in the paper it remains unclear how a generative model is specified in its entirety. Is what the user writes the equivalent of a likelihood or a simulator? Both pieces are important and can look like very distinct code (in fact, in most non-trivial cases, the simulator code and the likelihood code wouldn't resemble each other to the point where inferring one from the other seems impossible). Usually, it is the likelihood that underlies the objective function used for optimization and the simulator that is used for e.g. prior and posterior predictive checks.
The distinction is made only quite weakly in the text. "components" of models (e.g. in the cpm.models module) seem to refer specifically to the likelihood part. However arguably a "model" can reasonably be defined first and foremost as a simulator. From the flow chart in Fig 1., simulators seem to be associated only with the last step in the workflow. This has implications for the class of models that are considered a priori (see my points below concerning discussion toolboxes that focus on simulation-based inference, such as BayesFlow and the sbi toolbox in Python).

RESPONSE: We thank the reviewer for these suggestions. The confusion here is terminological, and the revised manuscript now states the user must specify trial-level computations that produce some predictive quantities and states the differences between generative and non-generative model specifications, both in the **Model Execution Framework (page 10, Line 290)**.
Briefly, the objective functions used in the optimisation routines are created from these predictions and the observed dependent variable. The model becomes generative when the predictive quantity, often a distribution, is paired with a response-generating mechanism, such as sampling from a distribution. This is handled at the Wrapper levels. We agree that a "model" can be defined as a pure simulator in some communities; in cpm, we use model specification to refer to the shared architecture that supports both likelihood-based fitting and simulation.

To improve clarity, we have now refined our phrasing in the **Implementation (page 6, Line 193)** section that the simulation is not restricted to the final stages of the modelling pipeline and can be used at any stage. The modelling workflow we outlined is just one example of what can be implemented in the toolbox. We have used it because it is relatively straightforward and underlies our example walkthrough.

Finally, we acknowledge that simulation-based inference toolboxes (e.g. BayesFlow) target different modelling approaches, cpm focuses on likelihood and loss-based estimation paired with model construction and forward simulation within a unified modelling interface. This is considered standard in our field and our terminology hopefully speaks well to the primary readership.

COMMENT#3: Second, the construction of a model in Listing (4) looks too much like ad-hoc code, from which it is hard to deduce specific principles about how to approach a user contribution. You might be

better served by adding a pseudo-code example that illustrates conceptual building blocks with less specific details.

RESPONSE: We thank the reviewer; this is a great suggestion. We have now included a checklist at the beginning of the **Specifying processes with cpm.models (page 19, line 649)** section in the **Building a model from scratch (page 18, line 625)** walkthrough and specified the fixed sequence of operations users must follow when writing the model function. To keep the walkthrough more straightforward, we included the recommended pseudocode in the **Supporting Information.** We included the pseudocode example below:

Algorithm 1 Delta-rule learner with softmax choice for a k-armed bandit

Require: Trials $\{(s_t, y_t, R_t)\}_{t=1}^{N}$, learning rate $\alpha \in [0, 1]$, inverse temperature $\beta \geq 0$
Ensure: Negative log-likelihood $\mathcal{L}(\theta \mid Y, M)$ with $\theta = \{\alpha, \beta\}$

Initialise $Q_1(a) \leftarrow 0$ for all actions $a \in \{1, \ldots, k\}$
$\mathcal{L} \leftarrow 0$
for $t = 1$ to N **do**
    $\mathcal{A}_t \leftarrow$ actions available on trial t ▷ e.g. $\{\text{left}, \text{right}\}$
    for each $a \in \mathcal{A}_t$ **do**   ▷ Softmax policy, Eq. (1)
        $u_t(a) \leftarrow \exp\big(\beta\, Q_t(a)\big)$
    end for
    $Z_t \leftarrow \sum_{a \in \mathcal{A}_t} u_t(a)$ ▷ Normalising constant
    for each $a \in \mathcal{A}_t$ **do**
        $P_t(a) \leftarrow u_t(a) \,/\, Z_t$
    end for
    $A_t \leftarrow y_t$ ▷ Action chosen by the participant
    $\mathcal{L} \leftarrow \mathcal{L} - \log P_t(A_t)$ ▷ Bernoulli/categorical NLL
    $\delta_t \leftarrow R_t - Q_t(A_t)$ ▷ Prediction error
    $Q_{t+1}(A_t) \leftarrow Q_t(A_t) + \alpha\, \delta_t$ ▷ Delta-rule update, Eq. (2)
    for each $a \neq A_t$ **do**
        $Q_{t+1}(a) \leftarrow Q_t(a)$ ▷ Unchosen values unchanged
    end for
end for
return $\mathcal{L}$

COMMENT#4: Third, (this is again emphasized below in response to a specific statement in the paper), the authors include too sparse a discussion of the existing software eco-system. Two axes should be explored and differences and similarities should be discussed in more detail. Axis one being similar tools with a priori somewhat similar goals, such as HSSM, EMC2. Axis two is a nod to adjacent toolboxes such as BayesFlow and sbi, which vastly expand the feasible mode space and provide a take on alternative overall modeling workflows. To aid readers in their approach to computational modeling in computational psychiatry and beyond, it is important to provide a sufficient level of contextualization along both axes.

RESPONSE: Thanks for these suggestions, which is also related to **COMMENT#7** made by **Reviewer 1**. We have now added a **Related Work (page 478, line 511)** section in the manuscript, where we discuss more clearly the differences in scope, end-to-end coverage, and general approaches between cpm and the toolboxes, all of which we now cite.

COMMENT#5: Fourth, too vague of a discussion of more technical details that leave the reader guessing. I will give two examples:

RESPONSE: We thank the reviewers for this suggestion. We have now expanded the technical details by separating the walkthrough from implementation and design details. See our response to **COMMENT#4** by **Reviewer 1.** We respond to your specific examples below.

1. The term “compiled” is used without a clear explanation of what is meant in this context. This ties in with the point below concerning differentiability.

RESPONSE: Thanks for pointing this out. We have made the operations clear and removed the term “compile”, which has a specific meaning in software engineering, such as compiling raw C code into an executable program; something we do not do here. We have also separated the technical details from the walkthrough, as mentioned in our response to **COMMENT#4** made by **Reviewer 1**, which we hope made the technical details clearer.

2. 2. The term “large-scale simulations” is used without explaining the core mechanic that allows the toolbox to assemble trial-wise code such that simulation is efficient downstream.

RESPONSE: We have now explained how trial-level data is processed and organised by the Wrapper in the new **Model Execution Framework (page 10, Line 290)**. and expanded on data requirements and organisation in the **Data Format and Processing section (page 11, Line 337)**.

COMMENT#6: Fifth, nowhere does the paper include a discussion of differentiability of likelihood functions. This is an important, de facto crucial concept for model construction and the scope of inference algorithms that can be applied downstream (sampling and/or optimization). E.g. can models be written and “compiled” (due proper clarification of the term as per above) as JAX function etc.. While it is not strictly necessary for this toolbox to be useful to the community, a discussion of this aspect through the lens of the ambition, extensibility of the toolbox etc. however is. I consider it crucial in the contemporary landscape of popular techniques in computational modeling as well as machine learning.

RESPONSE: Thank you for raising this point. We agree that differentiability of the likelihood (or, more generally, the objective function) is an important consideration because it constrains which inference algorithms are appropriate for parameter estimation. In line with the scope of cpm as a theory-driven cognitive modelling toolbox (rather than a machine-learning or differentiable-programming framework), cpm does not assume differentiability. Instead, our optimisation and inference routines are intentionally objective-agnostic: users either pick or provide a likelihood/loss function, and the choice of estimation method should be guided by its properties (e.g., smooth or differentiable vs. non-differentiable or simulation-based). We have added a short discussion (line 385) in the **Optimisation and Inference (page 12, line 370)** section clarifying this dependency. We also removed the word compile to avoid confusion, see our response to **COMMENT#5** above.

COMMENT#7: Sixth, relating to the standardization of workflows and reproducibility of computational experiments in computational psychiatry, as motivated in the introduction, the article could benefit from highlighting the specific features of the package that allow loading and saving of the models (eg. parameterization instantiations), meta-data (eg. task paradigm constants), experimental settings (eg. optimization settings), and results (beyond saving the traces or diagnostic figures).

RESPONSE: We agree that this is an important point. We introduced a **Reproducibility Utilities (page 6, line 178)** section that mentions how reproducibility is supported within an end-to-end computational workflow. Specifically, the section highlights: (i) export functions attached to both the parameter management system and optimisation routines, which serialise their state to plain-text tabular files; (ii)

parameter exports that record bounds and prior specifications alongside parameter values; (iii) optimisation exports that capture extensive metadata including warnings, Hessian matrices, goodness-of-fit metrics, iteration counts, and termination criteria; and (iv) the broader design principle that metadata is retained throughout the workflow wherever possible, so that analyses can be inspected, shared, and rerun with minimal ambiguity. Together, these features go beyond saving traces or diagnostic figures and address the specific archival concerns raised.

Built-in serialisation of models as Python objects is not yet supported and planned for a future release. Models in cpm are currently lightweight, code-defined objects, so for now we rely on standard version-control practices for these components while cpm's archival utilities focus on runtime-generated state (fitted parameters and optimisation metadata) that would otherwise be lost. Extending the export system to cover model instantiations directly is a natural next step and we are actively considering the most useful format.

COMMENT#8: Seventh, it is unclear whether the toolbox would support (or aims to support) incorporation of other task paradigms employed in computational psychiatry moving beyond the canonical learning or decision-making tasks - for example, trust games, cognitive control tasks, etc.

RESPONSE: Thank you for this comment. We clarified this point in the **Scope (page 3, line 67)** section. cpm does not hard-code a fixed set of task paradigms but is designed around a standard interface in which a model operates on trial-level information and returns predictions and latent variables. As a result, tasks beyond canonical computational use-cases can be supported, provided the models can be expressed in the fashion specified in the **Constructing the Model (page 17, line 584)** section. At the same time, we specify now that current ready-to-use applications in cpm.applications focus on a small set of model families driven by early user needs, but broader coverage is expected to be added.

COMMEN,T#9: Reaction to specific quotes:

1. The authors comment (Line 48-49), that "existing toolboxes that cover computational methods used in the field tend to be special-purpose, with a strong focus on one type of model and a lack of flexibility in model building". This cites HDDM, HBayesDM and Piray et. al. (2019). Two comments:
    a. First, the citations should be expanded to include other toolboxes such as EMC2, HSSM and RLSSM and PyDDM (which is mentioned somewhat later in the paper in a different context).
    b. Second, specifically the HSSM eco-system, which this reviewer is involved in developing, is explicitly designed to foster community contributions centered around new models into a shared repository (while other aspects of emphasis may differ). The stated limitation of toolboxes in the field should be somewhat attentenuated.

Concerning the first point, please expand the citations to provide a broader picture of the existing eco-system.

RESPONSE: We very much thank the reviewer for pointing that out. We addressed this in our new **Related Work (page 15, line 484)** section, which now cites a broader range of packages, including HDDM, HSSM, EMC2, PyDDM, hBayesDM, catlearn, BayesFlow, and sbi, organised into three categories (evidence-accumulation models, fixed task/model catalogues, and simulation-based inference frameworks). Second, we have removed the sentence on Line 48-49 to avoid overstating the limitations of existing toolboxes. In particular, the revised text and the expanded HSSM treatment in **Related Work** explicitly acknowledge that HSSM covers aspects of model construction and is designed to support

community contributions of new models, and we now frame the distinction between cpm and HSSM as one of scope (breadth across modelling traditions vs. depth within sequential sampling models) rather than as a limitation of HSSM

COMMENT#10: Concerning point two (which comes up in other comments above) please provide some level of description of the differences between what you are doing and what a tool like HSSM provides.

2. In lines 124-126 the authors claim that "a significant distinction between cpm and other tools employed in Computational Psychiatry is its capacity to assist users in both constructing models and estimating parameters… [...]" In a similar vein to the above comment concerning the sparse representation of the existing software eco-system, the authors should attenuate this claim and possibly contrast more closely with tools like HSSM (and it's wider eco-system) which do take these thoughts into account (albeit manifesting quite differently).

RESPONSE: We agree that the original claim was somewhat misleading. We have now included a direct comparison between HSSM and cpm in the **Related Work (page 15, line 478)** section to give a fuller treatment of HSSM in related libraries. We now make explicit that HSSM (and related libraries) is the appropriate choice for users working on sequential sampling models, and that cpm deliberately does not aim to duplicate this coverage. The distinction between cpm and HSSM is now framed as one of scope rather than capability: HSSM offers depth within the evidence-accumulation family with its own model-construction facilities, whereas cpm aims for breadth across the broader set of theory-driven modelling traditions used in computational psychiatry.

Minor Comments

RESPONSE: Thank you for the detailed line-by-line suggestions. Since initial submission, the paper has undergone substantial rewriting, the specific line numbers this reviewer references no longer correspond to the revised text. We have reviewed each comment and incorporated the copy-edits where the text remained unchanged. We have further clarified key terminology (explanation of stateful list processor and removed compile to avoid confusion with the specific software engineering meaning). We have refined scope and comparison to other toolboxes to avoid unintended claims of uniqueness. Finally, we specified what not "supporting DDMs" means in the current release, rather than implying it is a fundamental incompatibility. We also added appropriate citations.

Line by line:

Line 12:
"Using such an approach…" → "This approach"

RESPONSE: We thank you for this suggestion. We amended the text.

Line 20-22:
Run on sentence, could be formulated to be a bit more crisp

RESPONSE: If you meant the sentence beginning with "*Individual laboratories…*" on Line 20, then this sentence has a single independent clause, therefore is not a run-on sentence.

Line 31:
"For them" → too informal", and" → start new sentence

RESPONSE: Amended the text.

Line 46:
Quotation marks need to be corrected

RESPONSE: We have corrected them.

Line 54-:
“We believe that such an open-source open-access toolbox” → in light of major comments concerning the slight underrepresentation of the toolbox eco-system, I would consider it more appropriate to us phrasing as: “... such open-source open-access toolboxes” to highlight the general point without insinuating uniqueness as strongly

RESPONSE: We thank the reviewer for this point. We amended the text. In addition, we now introduced a new **Related Work** section to address the larger point about representation of the toolboxes.

Line 57-74:
The difference between the description of modularity and flexibility is somewhat weak. Flexibility seems to essentially follow from modularity here. Maybe flexibility is rather described as interoperability?

RESPONSE: After the major restructuring, the text you refer to is no longer part of the manuscript.

Line 65:
“The toolbox” → surrounding sentences use “the toolbox” too many times, try to reduce repetition

RESPONSE: Similarly to the above comment, after the major restructuring, the text you refer to is no longer part of the manuscript.

Line 79:
“have distinct purposes” → “have a distinct purpose”

RESPONSE: The text you are referring to has been moved to the **Implementation (page 6, Line 193)** section. We have amended the text there.

Line 108:
“with concrete example” → “with concrete examples” ?

RESPONSE: We have corrected the text.

Line 111:
“To any scientists/researchers” → … “in the domain of [...]”

RESPONSE: We changed the text to “Bandit tasks are widely used and familiar to many researchers in the general domain of cognitive science…”.

Line 114:
“[...], where” → make it new sentence

RESPONSE: We split the sentence into two.

Line 132-133:
“[...] that are implemented as stateful list processors” → Somewhat unmotivated terminology. Since “stateful list processors” comes up a few times, it might be worth actually explaining what exactly this concept is about.

RESPONSE: We thank the reviewer for this suggestion. And we agree that we could have designated more space to explain the term. The second paragraph in the **Core Components (page 8, line 218)** section now explains the concepts behind stateful list processors. On the other hand, the terminology is motivated through the approach we take in model implementation, which is based on an implementation methodology developed by catlean (Wills et al., 2018), an R package, where term was first introduced.

Line 144:
“In such cases, the toolbox compiles” → I was unable to dissect what exactly is meant by the term “compiled” in this paper. Is this the technical use of the term, as in “compiled code”?

RESPONSE: The text you are referring to has been moved to the **Model Execution Framework (page 10, line 290)**. We acknowledge the confusing use of the word “compiled”, which has a specific meaning in software engineering. We have no amended this throughout the manuscript. See our blanket response above.

Line 186:
“[...] their own models, either from scratch or [...]” → break up sentence

RESPONSE: The text you are referring to has been removed from the manuscript during the revision.

Line 260:
“encapsulates the model with Wrapper” → “encapsulated the model with the wrapper class”?

Wrapper is also a very generic term, possibly consider renaming the functionality.

RESPONSE: We thank the reviewer for this suggestion. We have updated the text accordingly. Regarding the term *Wrapper*, given the extensive codebase and its widespread usage, we decided to retain it to avoid introducing inconsistencies.

Line 266:
“Wrapper objects can also serve as breakpoints, [...]” → endpoints? Somewhat unusual phrase and specifically in the context of software, mostly used when talking about debugging.

RESPONSE: Thank you for this observation. We agree that *breakpoints* may be misleading in a software context. The intent is that Wrapper objects serve as points where users can pause their computational workflow to interact with other tools or experiment; and in principle they can stop to investigate outputs for debugging. We have revised the text to use *checkpoints*, which better reflects this more general purpose.

Line 287-289:
“[...], and are repurposed here to avoid codebase fragmentation that would lead to unnecessary divergence - more informally, we do not with to ‘reinvent the wheel’” → Can be formulated positive as e.g. “we use battle-tested code from established libraries that are part of the scientific computing stack in python”

RESPONSE: Thank you for the suggestion. We appreciate the alternative phrasing but have opted to retain the original formulation as we feel it effectively conveys the intent in a direct and accessible way.

Line 311:
“Cpm.optimisaion.compare” → typo

RESPONSE: We have corrected the text.

Line 324:
“into a hierarchical one” → “into a hierarchical counterpart”

RESPONSE: We have corrected the typo.

Line 325:
“The users have already defined [...]” → “a user was already prompted to …”

RESPONSE: The text has been amended, and can now be found in the **Hierarchical Estimation (page 12, line 370)** sub-section within the **Optimisation and Inference** section.

Line 326: Again, the use of the term “compiled” is a bit unclear.

RESPONSE: We removed the term throughout the manuscript.

Line 332:
“Data-driven techniques that directly estimate hyper-parameters for hierarchical models from empirical data, namely Hierarchical Bayesian Inference based on Variational Bayes [...]” → Slightly unclear use of terminology. In principle Variational Inference is not a “counterpart” to “Empirical Bayesian Estimation”. Variational Inference is just an optimization based approach to estimate probability distributions (posteriors in the standard sense of Bayesian Inference). It doesn’t imply anything specific about directly estimating “hyper-parameters” [...] from empirical data.

RESPONSE: Thank you for this clarification. We acknowledge the terminological imprecision. In our implementation, Variational Inference is specifically used to estimate group-level hyperparameters within the HBI framework, which we have clarified in the revised text.

Line 346:
“The Simulator class streamlines large-scale simulations, [...]” → “large-scale” is used too generically.

RESPONSE: We have changed it to “streamlines simulations with large sample sizes”.

Line 353:
“[...], which users can then use” → “[...], which can then be used” e.g.

RESPONSE: We made the correction.

Line 359:
“All of this was done without leaving the toolbox or relying on large amounts of error-prone code” → too vague

RESPONSE: Thank you for this comment. We have revised the sentence to be more specific about what the pipeline entails and the benefits of the unified workflow. The revised sentence now reads: 'The

entire pipeline — model construction, parameter estimation, hierarchical estimation, and simulation — was completed within a unified toolbox workflow, eliminating the need for extensive custom scripting and reducing the risk of implementation errors'.

Line 385:

"We want to make it clear that if you decide to contribute, our goal will be to help you make the best contribution possible" → drop sentence

RESPONSE: We have removed the sentence.

Line 387-389:

"We have had very positive experiences with people who contributed substantially to previous packages, often resulting in large contributions as part of collaborative research projects" → Should be reformulated as a contribution guideline, this sentence as is is more appropriate for a grant application than a paper.

RESPONSE: The text you are referring to has been removed from the manuscript during the revision.

Line 426-428:

"The toolbox currently does not aspire to cover Drift Diffusion Models, as these are already comprehensively supported by the Python library, PyDDM [...]" → Should cite HDDM, HSSM, EMC2 (and possibly more) here. Moreover, it is unclear what "not supporting" means in the context of the story of the paper. Not attempting to include Drift Diffusion Models as a core model with examples in the codebase? Or is there a fundamental notion as to how it would be difficult to support these models?

RESPONSE: Thank you for this comment. We have added citations to HDDM, HSSM, and EMC2 alongside PyDDM to better reflect the existing ecosystem. Regarding the lack of clarity around 'not supporting' DDMs, we have revised the text to make explicit that this refers to DDMs not being included as core models with dedicated examples in the codebase, rather than any fundamental incompatibility. As noted in our response to Reviewer#1, Comment#17, we view interoperability with existing DDM packages as the most sensible path forward, and we are currently exploring how such an implementation could be realised in future releases. We believe the revised **Related Work (page 15, line 478)** and **Scope (page 3, line 67)** section now better contextualises these decisions.

General:

Sometimes empty lines are used after e.g. imports in the codeblocks and sometimes not. Make formatting homogeneous on that front.

RESPONSE: We thank the reviewer for pointing this out. We have now made the format homogeneous.

Reviewer #3:

COMMENT#1: This paper introduces a toolbox, Computational Psychiatry Modeling Library or cpm, for carrying out cognitive computational modeling research. The paper does a good (albeit brief) job motivating why a package is necessary. The lack of generally available toolboxes means that most models are hand-fit which creates a barrier to entry into the field and increases the risk of bugs and other errors.

RESPONSE: We thank the reviewer for the positive feedback on the motivation behind the software. Following this comment, we have expanded the motivation by adding a related work section that positions cpm relative to existing toolboxes — what cpm offers that others do not is itself part of the justification for its existence. We have carefully addressed all remaining concerns below and hope the revised manuscript meets the reviewers' expectations.

COMMENT#2: 1. Lack of standardization

Authors' goal of growing this toolbox as an open-source project is laudable, but they should consider addressing how they will maintain stylistic consistency and code quality in such a project. How will they ensure that new features continue to work well together in the modular fashion they envision? While this question does not need to be directly answered, some consideration of these issues would be helpful to include.

RESPONSE: That is a very important point. We have clarified how the toolbox implements guardrails against code breakage and quality decline during third-party contributions. Specifically, we linked how testing units guard against code breakage resulting from change to the code in the new **Testing and Stability (page 14, line 463)** section; and added more details on how contributions are reviewed and checked through collaboration features on GitHub in the **Contribution (page 30, line 858)** section. Finally, we created (and pointed readers) to the CONTRIBUTING.md file on GitHub, which is a common way to outline contribution guidelines in open-source projects, such as ours. Note, that people cannot make direct changes to the code on GitHub, unless they are added as contributor to the repository by the maintainer (currently the first author).

COMMENT#3: Authors state that models were "hand-selected" (p. 3) for inclusion in toolbox. Authors should consider providing some explanation of the reasons for selecting these models.

RESPONSE: We thank the reviewer for this suggestion and agree the original wording was too vague. We also think it is an important and fair point. The **Scope (page 3, line 67)** now explains the rationale for the initial model selection. The included models were chosen to (i) cover modelling traditions widely used in computational psychiatry, (ii) span more than one theoretical tradition rather than concentrate within a single family (e.g. reinforcement learning and prospect-theoretic models), (iii) target areas not already well served by existing toolboxes, since evidence-accumulation models are extensively covered elsewhere, and (iv) include models with established empirical use cases so that new users can reproduce familiar analyses. Within these criteria, the order of implementation has also been responsive to user needs; for example, the prospect theory models were prioritised in response to early testers' requirements.

COMMENT#4: state that "each implemented model component has accompanying documentation with appropriate references" (p. 11). References were missing for some components. The documentation needs to be strengthened so that all toolbox components have accompanying documentation. For example, what choice models are implemented in the toolbox, and what are their formulas?

RESPONSE: Thank you for this suggestion. We have checked the references and formal specifications one by one and ensured that appropriate documentation exists for all. Following publication, requests for additional documentation and references can be submitted via the GitHub Issues platform, making the process transparent and community-driven.

COMMENT#5: 2. Lack of accessibility

It is not entirely clear what audience this paper is directed at, perhaps because this toolbox is intended to be useful for both beginners and experts in the field. The result is that portions of the paper do a good job of explaining concepts at the level of an interested beginner, but other portions shift into language/jargon that requires much more prior knowledge of the field to understand. One simple example is that authors could add a few sentences describing what participants do during the bandit task.

RESPONSE: Thank you for this suggestion. Reviewer 1 made the same point about accessibility, see our response to their **COMMENT#4** above**.** Briefly, this has been alleviated by separating the technical details from a didactic walkthrough. We also added more explanation on the bandit task in the **Result** section on page 16 were we now write:

"In a canonical bandit task, participants repeatedly choose between alternatives, often referred to as arms, to earn rewards, with the overall goal of maximising the cumulative rewards earned by the end of the experiment. Each option is associated with an underlying probability of generating a reward. So, if the stimulus has a corresponding reward rate of 80%, it–on average–results in a reward on 80% of the trials and in no reward on 20% of trials. In our current experiment, each trial presents two stimuli (arms) out of a battery of four, participants select one, and then receive binary feedback indicating reward or no reward."

COMMENT#6: A general question - why is this a computational psychiatry-focused toolbox? It seems that most of the models are from decision-making or cognitive science, and there is little to no treatment of how to handle individual difference questions when modeling.

RESPONSE: We agree. Computational Psychiatry largely builds on established models and inference techniques from Computational Cognitive Neuroscience and Cognitive Science, because they are the workhorse for generative models that can formalise latent mechanisms that can be mapped to psychiatric symptoms, groups, and clinical outcomes. We have clarified our position in the revised manuscript that cpm targets computational psychiatry use-cases and development is guided by the needs of the community, but the toolbox itself is not restricted to computational psychiatry and can be applied in related fields. See the **Introduction** section (line 61).

We also thank for your comment on individual differences. A similar comment (**COMMENT#8**) was made by **Reviewer 1**, see our response above. Briefly, we strengthened the treatment of individual differences by explicitly describing various analysis paths in the **Optimisation and Inference (page 12, line 370**) section.

COMMENT#7: 3. Lack of Flexibility

The toolbox is designed for computational psychiatry yet assumes that models will be fit to single subject behavior. Hierarchical modeling uses empirical Bayesian approaches which have major caveats by "double dipping" data. Given that that state-of-the-art in computational psychiatry is to use

hierarchical approaches, the toolbox does not sufficiently cover or handle this type of model fitting. This is a major concern as toolboxes often set methodological standards for the field.

RESPONSE: We appreciate the reviewer raising this methodological point. While early parts of the walkthrough focus on subject-level fitting, cpm does not assume that models must be fit independently to each participant. In particular, setting prior=True in the optimisation routines incorporates the user-specified priors into the objective function (Maximum A Posteriori (MAP)-style fitting, where the log-prior is added to the objective function for each subject). In addition, cpm supports hierarchical estimation through cpm.hierarchical, where group-level prior hyperparameters can be estimated from data using two implemented approaches.
We agree, however, that the current release does not aim to provide the full range of hierarchical Bayesian workflows (e.g., fully Bayesian MCMC-based inference).
This has been clarified in the **Hierarchical Estimation (page 12, line 370)** within the **Optimisation and Inference** section. We have also explicitly encouraged people to use priors in their parameter estimation in the **Hierarchical models with cpm.hierarchical (page 24, line 731)** section in the walkthrough.

Regarding the concern about double dipping: this issue is most problematic when the same data are used to both select a model and make inferences about it, inflating false positive rates. The two implemented methods use the data to estimate group-level priors – this is a known and accepted trade-off rather than a fundamental flaw. The implemented methods incorporate a measure of uncertainty via a Hessian-based approximation around MAP estimates, though we acknowledge these do not fully extend to hyperparameter uncertainty as in fully Bayesian workflows. A cross-validated hyperprior estimation scheme would further mitigate this concern and is a natural target for future contributions.

Additional code issues:

RESPONSE: We thank the reviewer for catching these issues. We have reviewed the code and fixed the issues reported. Since the first submission of the manuscript, the toolbox has undergone substantial development, including several new fixes due to changes in external dependencies, new Prospect Theory model categories, and several data processing utilities; a full list of changes is available in the changelog on GitHub [LINK]. To ensure that readers can run the walkthrough without dependency or environment issues, we have made the fully executable version available on Google Colab: [LINK]. The Colab notebooks mirror the manuscript walkthrough exactly and require no local setup.

(all run in jupyter notebook with python version 3.12.2 on MacOS Sequoia 15.7.1)
1. Code in paper
a. Parallelisation did not work in Listing 6 and 7 as written
a. Error: NameError: name 'cpm' is not defined
b. Some examples in cpm github that used parallel computing did run successfully, suggesting the issue is specific to the code samples included in the paper.
b. Simulation code in Listing 8 did not run correctly
a. Raised ValueError: the number of groups in the data and parameters should be equal.
2. Github examples
a. Could not reproduce same values in outputs in docs/examples/bandit-task.ipynb, possibly due to changes in bandit dataset.
b. Encountered errors docs/examples/fitting-hierarchical-estimation.ipynb (simulation section) and docs/examples/metacognition.ipynb (generating plots)
c. Consider finding a way to make simulation results fully replicable (setting a random seed?).
