# Supplementary material for: cpm: A python library for theory-driven modelling in computational psychiatry
Source: PLoS Comput Biol. 2026 Jul 13;22(7):e1014481. doi: 10.1371/journal.pcbi.1014481 (PMC13379104; doi:10.1371/journal.pcbi.1014481)
Supplement: Peer review history

# Peer review: Decision Letter 0

**DOI:** 10.1371/journal.pcbi.1014481.r001

**Authors:** Thomas Serre, Frederike Petzschner

18 Nov 2025

PCOMPBIOL-D-25-01874

cpm: a python library for theory-driven modelling incomputational psychiatry

PLOS Computational Biology

Dear Dr. Dome,

Thank you for submitting your manuscript to PLOS Computational Biology. After careful consideration, we feel that it has merit but does not fully meet PLOS Computational Biology's publication criteria as it currently stands. Therefore, we invite you to submit a revised version of the manuscript that addresses the points raised during the review process.

Please submit your revised manuscript by Jan 18 2026 11:59PM. If you will need more time than this to complete your revisions, please reply to this message or contact the journal office at ploscompbiol@plos.org. When you're ready to submit your revision, log on to https://www.editorialmanager.com/pcompbiol/ and select the 'Submissions Needing Revision' folder to locate your manuscript file.

Please include the following items when submitting your revised manuscript:

* A rebuttal letter that responds to each point raised by the editor and reviewer(s). You should upload this letter as a separate file labeled 'Response to Reviewers'. This file does not need to include responses to formatting updates and technical items listed in the 'Journal Requirements' section below.

* A marked-up copy of your manuscript that highlights changes made to the original version. You should upload this as a separate file labeled 'Revised Manuscript with Track Changes'.

* An unmarked version of your revised paper without tracked changes. You should upload this as a separate file labeled 'Manuscript'.

If you would like to make changes to your financial disclosure, competing interests statement, or data availability statement, please make these updates within the submission form at the time of resubmission. Guidelines for resubmitting your figure files are available below the reviewer comments at the end of this letter

We look forward to receiving your revised manuscript.

Kind regards,

Frederike Petzschner

Guest Editor

PLOS Computational Biology

Thomas Serre

Section Editor

PLOS Computational Biology

**Additional Editor Comments :**

Thank you very much for submitting your manuscript to PLOS Computational Biology. All reviewers agreed that your study makes a valuable and original contribution to the field. In particular, they commended the scientific motivation and potential impact.

At the same time, the reviewers identified several important issues that will need to be addressed before the manuscript can be considered for publication. The main concerns relate to (1) the clarity and completeness of the modeling description and its relation to existing approaches, and (2) difficulties encountered in running the provided code. We therefore invite you to submit a major revision that fully addresses all points made by the reviewers.

**Journal Requirements:**

1) We ask that a manuscript source file is provided at Revision. Please upload your manuscript file as a .doc, .docx, .rtf or .tex. If you are providing a .tex file, please upload it under the item type u2018LaTeX Source Fileu2019 and leave your .pdf version as the item type u2018Manuscriptu2019.

2) Please ensure that your article adheres to the standard Software article layout and order of Abstract, Introduction, Design and Implementation, Results, and Availability and Future Directions. For details on what each section should contain, see our Software article guidelines:

https://journals.plos.org/ploscompbiol/s/submission-guidelines#loc-software-submissions

3) Please upload all main figures as separate Figure files in .tif or .eps format. For more information about how to convert and format your figure files please see our guidelines:

https://journals.plos.org/ploscompbiol/s/figures

4) We notice that your supplementary information is included in the manuscript file. Please remove them and upload them with the file type 'Supporting Information'. Please ensure that each Supporting Information file has a legend listed in the manuscript after the references list.

Note: If the reviewer comments include a recommendation to cite specific previously published works, please review and evaluate these publications to determine whether they are relevant and should be cited. There is no requirement to cite these works unless the editor has indicated otherwise.

**Reviewers' comments:**

Reviewer's Responses to Questions

**Comments to the Authors:**

**Please note that one review is uploaded as an attachment.**

Reviewer #1: This manuscript presents cpm, a Python library for theory-driven computational modeling in psychiatry and cognitive neuroscience. The toolbox integrates a wide range of modeling approaches (reinforcement learning, associative learning, signal-detection, and hierarchical Bayesian estimation) in a unified framework intended for both novices and experts. The paper offers a walkthrough of cpm’s architecture, coding principles, model construction, optimization, hierarchical estimation, and simulation capabilities. I have co-reviewed this article with a graduate student in my lab.

On the whole, the toolbox addresses the important need in computational psychiatry to standardize model-fitting procedures and cut down on error-prone custom code across projects. Although we see considerable strength in the direction of the toolbox, the manuscript could be made stronger in terms of its didactic value and clarity, as well as its implementation. The current manuscript reads more like a developer’s overview than a didactic guide or validation paper. Clarifying the intended user, expanding empirical benchmarking, and restructuring the examples could greatly improve accessibility and impact.

We saw two problems in the construction of the paper: 1) the examples/listings do not flow in a linear way that would serve as a 'quick start' tutorial for a new user and give a sense of increasing complexity (from off-the-shelf to fully custom), and 2) the scope of models encompassed by cpm is not clear, making it hard for a reader to know if they should consider the package further.

In terms of the first concern, the example seeks to go from fully built to more customized, and this makes sense. But most of the transitions are stated in technical terms, like "We specify two free parameters: alpha for the learning rate, and a temperature parameter governing the choice stochasticity." Rather, such transitions should be explained more like, "Although we used default parameter values of X and Y for learning rate and temperature in the cpm.applications example above, what if we wish to specify different priors on the learning rate and temperature?" Similarly, when going to greater levels of complexity (e.g., specifying the model in Listing 4), the reader needs more of a checklist, like, "As the user moves from fully built models such as cpm.applications.reinforcement_learning.RLRW to more customized ones, they must specify the core ingredients of a model within the framework. In particular, as shown in Listing 4, the model definition must include..." Rather than stay the didactic level, the paper is too eager to communicate its breadth and novelty, like, "Thus, we put extra effort into these features of the toolbox, making the parameterisation of models within cpm robust, reliable, and equipped with numerous functionalities." To summarize, I strongly recommend separating a clear walkthrough of the RLRW/Q-learning model from efforts to describe the scope of the package and some of the interesting, but not directly relevant, architectural details. After the reader has a worked example in hand, including why they might wish to swap out the softmax choice rule for another, you can then transition to talking about things in more general terms, clarifying the architectural principles and major modules of cpm. At present, there are far too many asides in the example that distract, such as, "Examples include Q-values in reinforcement learning models [39], or transition matrices in model-based and model-free learning. These methods are often used internally by the library; for example, calculating the probability density functions (PDF) for priors is used to automatically turn any model into a hierarchical one without expecting the users to hand-code the transition."

Second, although we appreciate that it may be difficult to provide an exhaustive list of the packages scope, I believe you should try to try to include something on this matter. For example, consider including a table that lists the models available -- or even the major families. For example, there is already a clear “learning model” category inherent to the RLRW Wrapper. It would help the reader to spell out these categories more explicitly and give rationale and a plan for including categories in the future as the package expands. Likewise, what are the 'Legos' that users can reach for when building models and what will they have to code by hand? Without some exposition of the scope (it's currently rather promissory in the text), interest in the package may be limited.

At the conceptual level, our biggest concern is that the novelty of cpm relative to prior efforts is somewhat unclear. I believe the novelty lies in the modular approach. I also found the prior = True automation intriguing and novel. Although you emphasize these, there are no direct comparisons to prior efforts (e.g. hBayesDM), including areas where those efforts may be preferable for some applications. For example, hBayesDM gives full MCMC for many common off-the-shelf models where applied users may have no particular motivation to innovate. Consider including a comparison table or summary in terms of package scope, flexibility/modularity, and estimation and model comparison functionality. More broadly, provide a clearer justification of how cpm can improve reproducibility and model comparability across labs compared to prior packages.

Second, although the toolbox has "computational psychiatry" in the title, it was unclear to us why this is so. Some useful justification is provided by, "that researchers – especially in Computational Psychiatry - estimate model parameters for individuals as opposed to group-level data." Yet more broadly, the toolbox contain many theory-driven cognitive computational models. To make it truly CP-specific, we would have anticipated new tools or methods for individual difference analysis. For example, some studies have considered whether to fit parameters by clinical group (e.g. Mkrtchian et al., 2017), while others have considered how best to compare parameter estimates across groups or how to link computational parameter estimates with more traditional self-report measures of symptoms or traits. cpm seems explicitly focused on within-person processes, not between-person differences in those processes. Greater clarity on the choice of naming and the omission of individual difference analysis tools would be welcome. Are there plans to develop some individual difference analysis tools that would make it relevant to CP per se?

At the technical/implementation level, I had a few suggestions as well. First, it would be helpful for to clarify whether cpm implements unit tests to avoid regression bugs and related issues. Relatedly, does cpm run benchmark tests of results against established implementations of the same model (e.g., in hBayesDM)? These can provide reassurances for the user that each of the core models in the package are trustworthy even as the cpm enables more customization (I appreciate that it would be impossible to create tests for all combinations).

Second, the details of the hierarchical prior=True implementation are somewhat unclear. The paper states "During the parameterisation of the model, the users have already defined priors for their parameters, so setting prior=True in the optimisation functions will allow the model to be compiled into a hierarchical model." Although we indeed see information on priors in Listing 3, very little was said about the selection of priors (a big topic, so I'm not suggesting you need to be comprehensive) and it was unclear how these enter into the hierarchical modeling. For example, the Piray HBI approach is rather different from traditional hierarchical modeling in that parameter estimates are informed primarily by those likely to belong to/be described by a given model in the set. Please provide additional detail about how prior=True affects model implementation and how this differs between estimation algorithms.

Finally, one of the biggest pain points for users adopting a new package is understanding its data structure expectations -- for example, what should this matrix contain, does that have to be a column vector, do I have to specify a lower bound, will the code blow up if I provide doubles instead of integers? Very little is said about this, yet the code listings reveal what could be strict expectations (e.g., .astype(int) in the stimulus definition of Listing 4). Likewise, several structure-related Pythonisms/conventions are embedded in the listings but not described. For example, the .flatten() method in Listing 4, or the .copy of Qvalues (Listing 4), or .groupby('ppt') in Listing 6. Please provide more guidance on the key inputs to the package and whether the package is tolerant of variation (e.g., whether it would convert to int internally for a multinomial outcome etc.)

Additional comments:

1. For applied users without a deep background in mathematics and programming (which cpm intends to serve), there seems like a large gap between using a pre-defined Wrapper and building a model from scratch. While the package provides basic functions for the latter, the code in Listing 4 is still somewhat tedious and may be inaccessible to many users. Is there a way to find a middle ground? For example: the lines in Listing 4 that call pre-defined functions in the package are clearly the important “modular” elements that are meant to be the heart of the package (e.g., `DeltaRule` versus `SeparableRule`, etc.). Could the developers add an argument to the RLRW Wrapper such as “learning_rule” that then the user could specify which of those sub-functions is being called? This would give the user the flexibility you seek but keep the non-computational user from having to copy and paste the base code and edit it.

2. pg. 7 The RLRW abbreviation is never defined.

3. Listing 4, Line 28-30 Why isn’t this done by the function or at least given as an option through an argument to the function? (Pertains to broader questions about data structure and tolerance of violations)

4. Although we appreciate the importance of not being redundant with other packages, not including DDM in some form seems like a weakness. Is there a way to wrap the PyDDM package into this one? Since you emphasize standardization/a unified approach I see this as being an opportunity to bridge the divide between DDM modelers and others in terms of software development, etc. Relatedly, is there support for RL-DDM approaches?

5. Model comparison is largely set to the side as a separate topic, but could potentially be incorporated into the walkthrough. On p. 10, this seems like a place to segue into the description of model comparison, "In this case, the generic cpm.models.learning.Softmax class includes this version as an associated method, so we just exchange choice_rule.compute() on lines 24 to choice_rule.irreducible_noise(). This allows users to explore how thematically related variants of the same computation impact model fits and behaviours. In terms of general software architecture, this approach lets cpm organise these modifications as part of the same class for ease-of-use and theoretical alignment"

Reviewer #2: The review is attached in a file.

Reviewer #3: This paper introduces a toolbox, Computational Psychiatry Modeling Library or cpm, for carrying out cognitive computational modeling research. The paper does a good (albeit brief) job motivating why a package is necessary. The lack of generally available toolboxes means that most models are hand-fit which creates a barrier to entry into the field and increases the risk of bugs and other errors.

Authors identify three problems:

1. Lack of standardization

o Leading to inconsistent results

o Errors in code

o Bespoke approaches from different lab that are suited only for specific purposes

2. Lack of accessibility for non-computational researchers

3. Lack of flexibility in existing approaches

Authors partially address the problems they identify. However, there are areas in which the answers authors propose could be stronger:

1. Lack of standardization

a. Authors’ goal of growing this toolbox as an open-source project is laudable, but they should consider addressing how they will maintain stylistic consistency and code quality in such a project. How will they ensure that new features continue to work well together in the modular fashion they envision? While this question does not need to be directly answered, some consideration of these issues would be helpful to include.

b. Authors state that models were “hand-selected” (p. 3) for inclusion in toolbox. Authors should consider providing some explanation of the reasons for selecting these models.

c. Authors state that “each implemented model component has accompanying documentation with appropriate references” (p. 11). References were missing for some components. The documentation needs to be strengthened so that all toolbox components have accompanying documentation. For example, what choice models are implemented in the toolbox, and what are their formulas?

2. Lack of accessibility

a. It is not entirely clear what audience this paper is directed at, perhaps because this toolbox is intended to be useful for both beginners and experts in the field. The result is that portions of the paper do a good job of explaining concepts at the level of an interested beginner, but other portions shift into language/jargon that requires much more prior knowledge of the field to understand. One simple example is that authors could add a few sentences describing what participants do during the bandit task.

b. A general question - why is this a computational psychiatry-focused toolbox? It seems that most of the models are from decision-making or cognitive science, and there is little to no treatment of how to handle individual difference questions when modeling.

3. Lack of flexibility

a. The toolbox is designed for computational psychiatry yet assumes that models will be fit to single subject behavior. Hierarchical modeling uses empirical Bayesian approaches which have major caveats by “double dipping” data. Given that that state-of-the-art in computational psychiatry is to use hierarchical approaches, the toolbox does not sufficiently cover or handle this type of model fitting. This is a major concern as toolboxes often set methodological standards for the field.

Additional code issues:

(all run in jupyter notebook with python version 3.12.2 on MacOS Sequoia 15.7.1)

1. Code in paper

a. Parallelisation did not work in Listing 6 and 7 as written

a. Error: NameError: name ‘cpm’ is not defined

b. Some examples in cpm github that used parallel computing did run successfully, suggesting the issue is specific to the code samples included in the paper.

b. Simulation code in Listing 8 did not run correctly

a. Raised ValueError: the number of groups in the data and parameters should be equal.

2. Github examples

a. Could not reproduce same values in outputs in docs/examples/bandit-task.ipynb, possibly due to changes in bandit dataset.

b. Encountered errors docs/examples/fitting-hierarchical-estimation.ipynb (simulation section) and docs/examples/metacognition.ipynb (generating plots)

c. Consider finding a way to make simulation results fully replicable (setting a random seed?).

**********

**Have the authors made all data and (if applicable) computational code underlying the findings in their manuscript fully available?**

The PLOS Data policy requires authors to make all data and code underlying the findings described in their manuscript fully available without restriction, with rare exception (please refer to the Data Availability Statement in the manuscript PDF file). The data and code should be provided as part of the manuscript or its supporting information, or deposited to a public repository. For example, in addition to summary statistics, the data points behind means, medians and variance measures should be available. If there are restrictions on publicly sharing data or code —e.g. participant privacy or use of data from a third party—those must be specified.

Reviewer #1: Yes

Reviewer #2: Yes

Reviewer #3: Yes

**********

PLOS authors have the option to publish the peer review history of their article (what does this mean?). If published, this will include your full peer review and any attached files.

If you choose “no”, your identity will remain anonymous but your review may still be made public.

**Do you want your identity to be public for this peer review?**  For information about this choice, including consent withdrawal, please see our Privacy Policy.

Reviewer #1: No

Reviewer #2: **Yes:** Alexander Fengler

Reviewer #3: No

[NOTE: If reviewer comments were submitted as an attachment file, they will be attached to this email and accessible via the submission site. Please log into your account, locate the manuscript record, and check for the action link "View Attachments". If this link does not appear, there are no attachment files.]

**Figure resubmission:**

While revising your submission, we strongly recommend that you use PLOS’s NAAS tool (https://ngplosjournals.pagemajik.ai/artanalysis) to test your figure files. NAAS can convert your figure files to the TIFF file type and meet basic requirements (such as print size, resolution), or provide you with a report on issues that do not meet our requirements and that NAAS cannot fix.

After uploading your figures to PLOS’s NAAS tool - https://ngplosjournals.pagemajik.ai/artanalysis, NAAS will process the files provided and display the results in the "Uploaded Files" section of the page as the processing is complete. If the uploaded figures meet our requirements (or NAAS is able to fix the files to meet our requirements), the figure will be marked as "fixed" above. If NAAS is unable to fix the files, a red "failed" label will appear above. When NAAS has confirmed that the figure files meet our requirements, please download the file via the download option, and include these NAAS processed figure files when submitting your revised manuscript.

**Reproducibility:**

To enhance the reproducibility of your results, we recommend that authors of applicable studies deposit laboratory protocols in protocols.io, where a protocol can be assigned its own identifier (DOI) such that it can be cited independently in the future. Additionally, PLOS ONE offers an option to publish peer-reviewed clinical study protocols. Read more information on sharing protocols at https://plos.org/protocols?utm_medium=editorial-email&utm_source=authorletters&utm_campaign=protocols

Attachment Submitted filename: ploscompbio-cpm-paper-review_final.pdf

# Author comment: Author response to Decision Letter 1

**DOI:** 10.1371/journal.pcbi.1014481.r002

24 Apr 2026

Attachment Submitted filename: Rebuttal Letter.pdf

# Peer review: Decision Letter 1

**DOI:** 10.1371/journal.pcbi.1014481.r003

**Authors:** Thomas Serre, Frederike Petzschner, Thomas Serre, Frederike Petzschner

22 Jun 2026

Dear Dr Dome,

We are pleased to inform you that your manuscript 'cpm: a python library for theory-driven modelling in

computational psychiatry' has been provisionally accepted for publication in PLOS Computational Biology.

Before your manuscript can be formally accepted you will need to complete some formatting changes, which you will receive in a follow up email. A member of our team will be in touch with a set of requests.

Please note that your manuscript will not be scheduled for publication until you have made the required changes, so a swift response is appreciated.

IMPORTANT: The editorial review process is now complete. PLOS will only permit corrections to spelling, formatting or significant scientific errors from this point onwards. Requests for major changes, or any which affect the scientific understanding of your work, will cause delays to the publication date of your manuscript.

Should you, your institution's press office or the journal office choose to press release your paper, you will automatically be opted out of early publication. We ask that you notify us now if you or your institution is planning to press release the article. All press must be co-ordinated with PLOS.

Thank you again for supporting Open Access publishing; we are looking forward to publishing your work in PLOS Computational Biology.

Best regards,

Frederike Petzschner

Guest Editor

PLOS Computational Biology

Thomas Serre

Section Editor

PLOS Computational Biology

***********************************************************

Reviewer's Responses to Questions

**Comments to the Authors:**

**Please note here if the review is uploaded as an attachment.**

Reviewer #1: I appreciate the substantial effort that has gone into restructuring the manuscript and addressing the reviewers’ comments. The paper is now considerably clearer in its intended audience and organization, with a much stronger tutorial flow, improved discussion of scope and related software, and substantially expanded explanations of the hierarchical estimation framework and implementation details. I also liked the new on testing, reproducibility, and software maintenance, as these strengthen confidence in the toolbox and its long-term development. While some limitations remain (e.g., the absence of formal benchmarking against existing implementations), the authors have been transparent about these points and have meaningfully improved both the manuscript and the presentation of the software. I think this paper and the cpm toolbox will be of considerable interest to the computational cognitive modeling and psychiatry communities.

Reviewer #2: I consider the revisions sufficient to address the main critiques I presented during the first round of review.

I want to thank the authors for addressing, specifically, comments on the overall structure of the paper, to streamline the presentation and improve didactic elements.

I caught two more small things while reading through the new version:

L222-231: These are essentially implemented, widely used-user equations → weird constructions, maybe something like “these are ‘pre-implemented, widely-used [...]”

L241: state-list processors → below occurs as “stateful list processors”, is this intended?

Reviewer #3: I thank the authors for their responsive revisions. They have addressed my comments from the first round of reviews.

**********

**Have the authors made all data and (if applicable) computational code underlying the findings in their manuscript fully available?**

The PLOS Data policy requires authors to make all data and code underlying the findings described in their manuscript fully available without restriction, with rare exception (please refer to the Data Availability Statement in the manuscript PDF file). The data and code should be provided as part of the manuscript or its supporting information, or deposited to a public repository. For example, in addition to summary statistics, the data points behind means, medians and variance measures should be available. If there are restrictions on publicly sharing data or code —e.g. participant privacy or use of data from a third party—those must be specified.

Reviewer #1: Yes

Reviewer #2: Yes

Reviewer #3: None

**********

PLOS authors have the option to publish the peer review history of their article (what does this mean?). If published, this will include your full peer review and any attached files.

If you choose “no”, your identity will remain anonymous but your review may still be made public.

**Do you want your identity to be public for this peer review?**  For information about this choice, including consent withdrawal, please see our Privacy Policy.

Reviewer #1: **Yes:** Michael Hallquist

Reviewer #2: **Yes:** Alexander Fengler

Reviewer #3: No

# Editor report: Acceptance letter

**DOI:** 10.1371/journal.pcbi.1014481.r004

**Authors:** Thomas Serre, Frederike Petzschner, Thomas Serre, Frederike Petzschner

PCOMPBIOL-D-25-01874R1

cpm: a python library for theory-driven modelling in

computational psychiatry

Dear Dr Dome,

I am pleased to inform you that your manuscript has been formally accepted for publication in PLOS Computational Biology. Your manuscript is now with our production department and you will be notified of the publication date in due course.

The corresponding author will soon be receiving a typeset proof for review, to ensure errors have not been introduced during production. Please review the PDF proof of your manuscript carefully, as this is the last chance to correct any errors. Please note that major changes, or those which affect the scientific understanding of the work, will likely cause delays to the publication date of your manuscript.

Soon after your final files are uploaded, unless you have opted out, the early version of your manuscript will be published online. The date of the early version will be your article's publication date. The final article will be published to the same URL, and all versions of the paper will be accessible to readers.

For Research, Software, and Methods articles, you will receive an invoice from PLOS for your publication fee after your manuscript has reached the completed accept phase. If you receive an email requesting payment before acceptance or for any other service, this may be a phishing scheme. Learn how to identify phishing emails and protect your accounts at https://explore.plos.org/phishing.

Thank you again for supporting PLOS Computational Biology and open-access publishing. We are looking forward to publishing your work!

With kind regards,

Sharmila Kamatchi

PLOS Computational Biology | Carlyle House, Carlyle Road, Cambridge CB4 3DN | United Kingdom ploscompbiol@plos.org | Phone +44 (0) 1223-442824 | ploscompbiol.org | @PLOSCompBiol
